# A Cascade Recognition of Activatable Probe for Fluorescence Navigation Glioblastoma Surgery: Overcoming Blood‐Brain Barrier and Off‐Target Limitations

**DOI:** 10.1002/advs.202521404

**Published:** 2026-02-10

**Authors:** Xinru An, Yu Guo, Yongning Bian, Yanli Tan, Chuan Fang, Hengzhu Zhang, Xueqian Chen, Lin Yuan, Dongdong Su

**Affiliations:** ^1^ Department of Chemistry Beijing University of Technology Beijing P. R. China; ^2^ Department of Neurosurgery Northern Jiangsu People's Hospital Yangzhou P. R. China; ^3^ Department of Neurosurgery Baoding No.1 Central Hospital Baoding P. R. China; ^4^ Hebei Key Laboratory of Precise Diagnosis and Treatment of Glioma Baoding P. R. China; ^5^ Department of Neurosurgery Baoding No.1 Central Hospital Baoding P. R. China; ^6^ College of Chemistry and Chemical Engineering Hunan University Changsha P. R. China; ^7^ Department of Neurosurgery Northern Jiangsu People's Hospital Affiliated to Yangzhou University Yangzhou China; ^8^ Department of Pathology Affiliated Hospital of Hebei University Baoding China

**Keywords:** activatable probe, blood‐brain barrier penetration, fluorescence navigation surgery, fluorescent probe, tumor margin delineation

## Abstract

Precise imaging and complete resection of glioblastoma (GBM) remain critically challenging due to two major obstacles: the inefficient delivery of probes across the blood‐brain barrier (BBB) and the lack of tumor‐specific activation, leading to poor contrast and inadequate tumor margin delineation. To address these challenges, we developed ANG‐hCy‐MC, a cascade recognition of activatable probe designed for high‐fidelity visualization of GBM. This probe leverages Angiopep‐2‐mediated targeting of LRP1 receptors to facilitate efficient BBB crossing, followed by a cascaded activation process triggered specifically by the tumor‐associated enzymes cathepsin B (Cat B) and monoamine oxidase (MAO). This dual‐enzyme cascade mechanism ensures ultra‐selective fluorescence turn‐on exclusively within tumor cells, thereby eliminating off‐target signals and providing exceptional tumor‐to‐normal tissue contrast, enabling the precise navigation and resection of orthotopic glioblastoma in live mice. Notably, in ex vivo human GBM specimens, ANG‐hCy‐MC achieved a remarkable tumor‐to‐normal fluorescence ratio of 7.83 at the invasive edge, enabling clear identification of even single infiltrating tumor cells. This probe allows real‐time, high‐contrast intraoperative guidance with unprecedented resolution, offering a powerful and clinically translatable strategy for achieving complete tumor resection and improved patient outcomes in GBM surgery.

## Graphical abstract

A cascade recognition of activatable probe ANG‐hCy‐MC is used for high‐precision depiction of glioblastoma (GBM) margin. This probe crosses the blood‐brain barrier (BBB) through LRP1 receptor‐mediated transport and provides excellent fluorescence signal contrast between the tumor and normal tissues, thereby enabling ultra‐selective imaging and precise resection of invasive GBM.

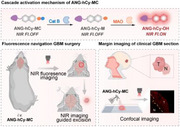

## 1 Introduction

Glioblastoma (GBM) is the most common and aggressive primary malignant brain tumor in adults. Maximizing the extent of safe surgical resection is a cornerstone of treatment, directly correlated with improved patient survival and prognosis [1, 2]. However, the diffuse infiltration of GBM cells into surrounding brain parenchyma makes achieving complete resection extraordinarily difficult without damaging critical neurological structures [3]. Consequently, the precise intraoperative delineation of tumor margins is one of the most critical yet unresolved challenges in GBM surgery [4]. Critically, conventional diagnostic techniques, including imaging modalities such as magnetic resonance imaging (MRI) and computed tomography (CT), as well as invasive biopsy samples for histopathological analysis, suffer from limitations such as procedural complexity, limited sensitivity, radiation exposure, and inherent invasiveness [5, 6]. Consequently, developing innovative imaging techniques to resolve tumor margin ambiguity remains imperative for effective GBM management. Fluorescence navigation surgery has emerged as a powerful alternative, overcoming many of these drawbacks by providing surgeons with real‐time, high‐resolution visual guidance to discriminate between malignant and healthy tissue [7, 8]. Clinically, fluorescence navigation surgery has been shown to enhance tumor resection completeness and improve patient survival [9]. This approach is particularly valuable in glioma surgery, where incomplete resection increases recurrence rates and negatively impacts survival, while excessive removal can result in neurological deficits and cognitive impairment [10]. By enabling intraoperative delineation of tumor boundaries and detection of otherwise occult lesions [11, 12], fluorescence navigation surgery represents a promising strategy to overcome the limitations of conventional imaging and address the critical challenge of accurate margin visualization [13, 14]. However, the efficacy of fluorescence navigation surgery is ultimately dependent on the performance of the fluorescent probe employed. Conventional fluorescent probes, typically activated by a single enzyme, are fundamentally limited by off‐target activation [15]. This lack of specificity causes false‐positive signals in healthy tissues, compromising surgical precision and potentially resulting in unnecessary resection [16, 17, 18].

To overcome this issue, dual‐lock activated fluorescent probes have been developed in recent years. It utilizes biomarkers to cooperatively recognize the fluorescence activation to achieve precise localization of tumor boundaries. Among them, the cascaded activated dual‐lock fluorescent probe combines the signal processing capability of “cascaded activation” with the high specificity of the “dual‐lock” logic gate control. This design strategy has a more outstanding ability to distinguish between tumor cells and normal cells, effectively suppressing false positive signals from living metabolic organs. The efficacy of this strategy relies on identifying biomarker pairs that are specifically co‐expressed in GBM and can participate in a cascade activation process [19]. In GBM, the co‐expression of cathepsin B (Cat B), a lysosomal cysteine protease, and monoamine oxidase (MAO), a key enzyme in neurotransmitter metabolism, offers an ideal pair for such an approach, and a large number of existing studies have fully confirmed that Cat B and MAO are highly expressed in clinical GBM, and both play significant roles in the development, invasion, and poor prognosis of the tumor [20, 21]. Although Cat B alone lacks sufficient specificity, its combination with MAO, which exhibits marked overexpression in GBM compared to normal brain [22, 23], enables a two‐step cascade activation mechanism that minimizes off‐target signals and allows precise delineation of tumor boundaries [24, 25]. This dual‐enzyme cascade activation strategy markedly improves tumor margin recognition [26], demonstrating strong potential to robustly distinguish tumor from normal tissue and providing a critical foundation for developing highly reliable navigational probes to guide GBM resection.

Beyond the challenge of specificity, the effective delivery of probes to the tumor site presents another major hurdle. The blood‐brain barrier (BBB) effectively blocks over 98% of systemically administered drugs from entering the brain [27, 28, 29]. To overcome this, receptor‐mediated transcytosis (RMT) via low‐density lipoprotein receptor‐related protein 1 (LRP1) offers a promising route for enhancing brain delivery [30, 31, 32]. LRP1, a key transporter at the BBB, mediates the transcellular transport of macromolecules while preserving BBB integrity [33]. Another study has shown that LRP1 is expressed in the cytoplasm and on the cell surface of glioma cells, thus having the ability to target glioma cells [34]. Harnessing this dual biological functionality, the targeting peptide Angiopep‐2 (ANG), a peptide with high affinity and specificity for LRP1, provides a promising solution [35]. Therefore, diagnostic probes conjugated with ANG exploit LRP1‐mediated transcytosis, substantially enhancing BBB penetration and facilitating GBM‐specific accumulation within the brain [36].

**SCHEME 1:**
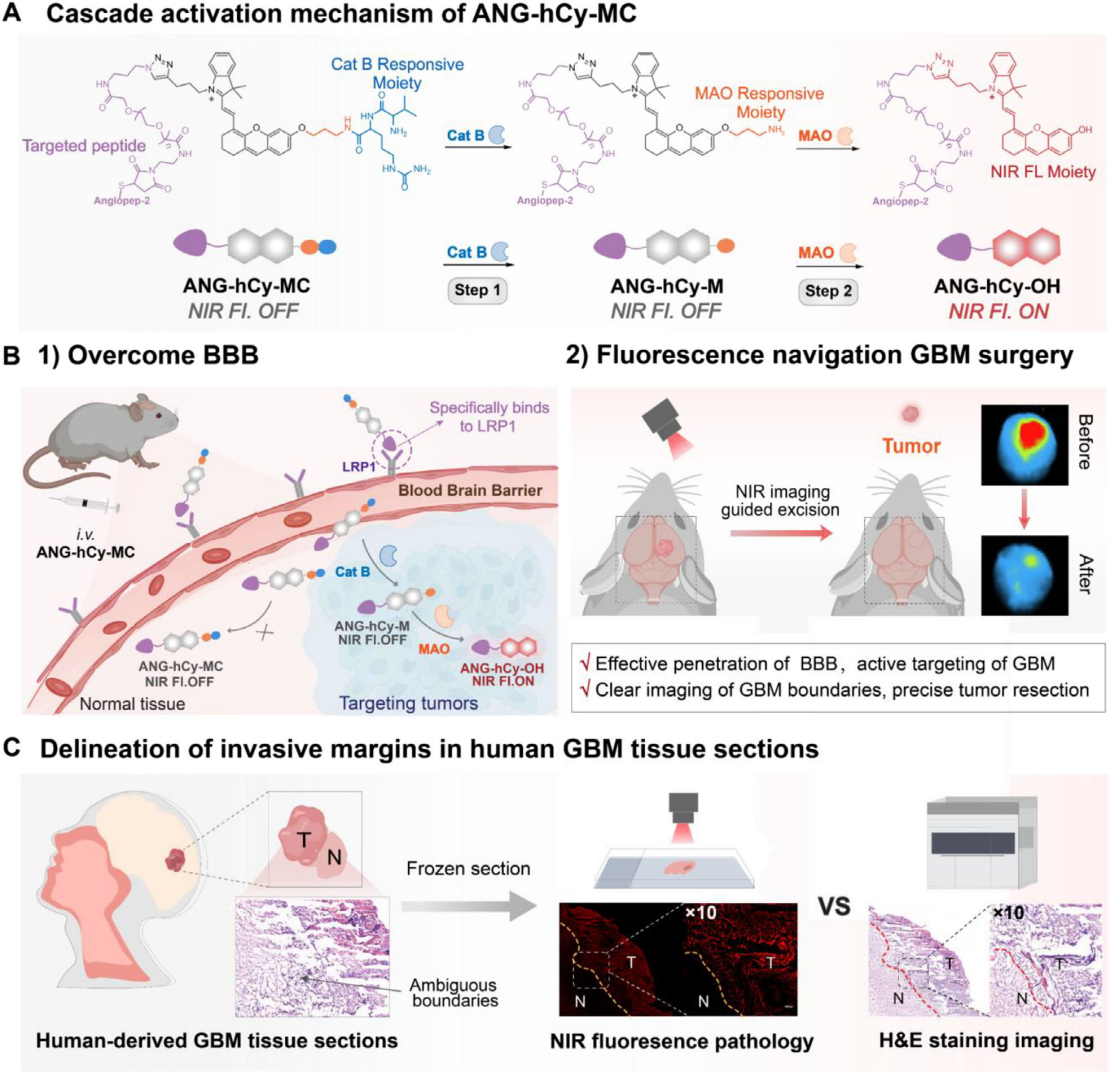
Cascade activation design strategy and biomedical application of ANG‐hCy‐MC for GBM detection. (A) Chemical structure and cascade activation mechanism of ANG‐hCy‐MC. (B) 1) Schematic diagram of penetrating the BBB and activation process. 2) Fluorescence navigation surgical resection. (C) Delineation of invasive margins in human GBM tissue sections.

To simultaneously overcome the dual challenges of BBB penetration and tumor‐specific activation, we engineered ANG‐hCy‐MC. This innovative probe integrates: (1) an Angiopep‐2 (ANG) motif enabling LRP1‐mediated transcytosis across the BBB and into GBM cells; (2) a tandem enzyme‐responsive substrate for sequential activation by Cat B and MAO, forming a cascade “dual‐lock” mechanism that ensures tumor‐restricted fluorescence activation; and (3) a near‐infrared (NIR) fluorophore facilitating deep‐tissue imaging (Scheme 1A). The probe operates through a sequential two‐step enzymatic cascade: initial cleavage by Cat B followed by MAO‐triggered dealkylation. This sequence confines fluorescence turn‐on exclusively to tumor microenvironments co‐expressing both enzymes, drastically reducing off‐target signals and enabling precise delineation of the GBM margin. It has been successfully applied in the guided surgical resection using NIR imaging in orthotopic mouse GBM model (Scheme 1B). Additionally, in human GBM tissue sections, ANG‐hCy‐MC achieved excellent tumor‐normal tissue distinction at the clinical GBM invasive margin (Scheme 1C). Compared with H&E staining, the NIR fluorescence pathology using ANG‐hCy‐MC can clearly delineate the tumor (T) and normal (N) tissue interface at the cellular resolution. This study combines the ANGmediated BBB penetration with the cascade activation strategy of Cat B/MAO for GBM surgical navigation. By synergizing the enhanced BBB penetration with the strict amplification of the dual‐enzyme cascade signal, ANG‐hCy‐MC establishes a transformative and clinically promising platform to achieve precise resection of GBM.

## 2 Results and Discussion

### 2.1 Rational Design and Synthesis of ANG‐hCy‐MC

The hemicyanine‐based fluorophore (hCy‐OH) was employed as the fluorescent scaffold [37]. The MAO‐responsive substrate (*tert*‐butyl *N*‐(3‐bromopropyl) carbamate) was conjugated to hCy‐OH via nucleophilic substitution reaction, followed by deprotection to afford intermediate hCy‐M. This moiety was subsequently functionalized with a Cat B‐cleavable substrate (Boc‐Val‐Cit‐OH) through amide condensation [38, 39]. Final deprotection yielded the core fluorescent unit hCy‐MC, bearing dual‐enzyme cascade responsive elements. ANG conjugation was achieved via copper(I)‐catalyzed azide‐alkyne cycloaddition (CuAAC), resulting in the final product ANG‐hCy‐MC. To systematically assess the targeting specificity and sequential activation behavior of our cascade recognition of activatable probe, we designed and synthesized ANG‐hCy‐MC along with two critical control probes: the mono‐enzyme responsive probe ANG‐hCy‐M (activated solely by MAO) and the non‐targeting counterpart PEG‐hCy‐MC (lacking LRP1 affinity) (Schemes S1 and S2). This strategic design allows direct evaluation of both the BBB traversal capability and the dual‐enzyme cascade activation mechanism. Ultraviolet‐visible absorption spectroscopy confirmed successful structural modifications: ANG‐hCy‐MC (ANG‐hCy‐M) exhibited a pronounced absorption increase at ∼250 nm compared to hCy‐MC (hCy‐M), confirming successful ANG peptide conjugation (Figure S1A,C), while PEG‐hCy‐MC showed a characteristic absorption peak at ∼230 nm, indicative of PEG incorporation (Figure S1B). Next, zeta potential measurements revealed the surface charge characteristics of hCy‐MC and the final product ANG‐hCy‐MC in aqueous solution, which was also observed for ANG‐hCy‐M, confirms the successful attachment of the targeting peptide (Figure S2). Key compounds were characterized using high‐resolution mass spectrometry (HRMS) and nuclear magnetic resonance spectroscopy (^1^H NMR and ^13^C NMR) (Figures S22‐S33).

### 2.2 Spectroscopic Validation of the Sequential Enzymatic Activation of ANG‐hCy‐MC

**FIGURE 1:**
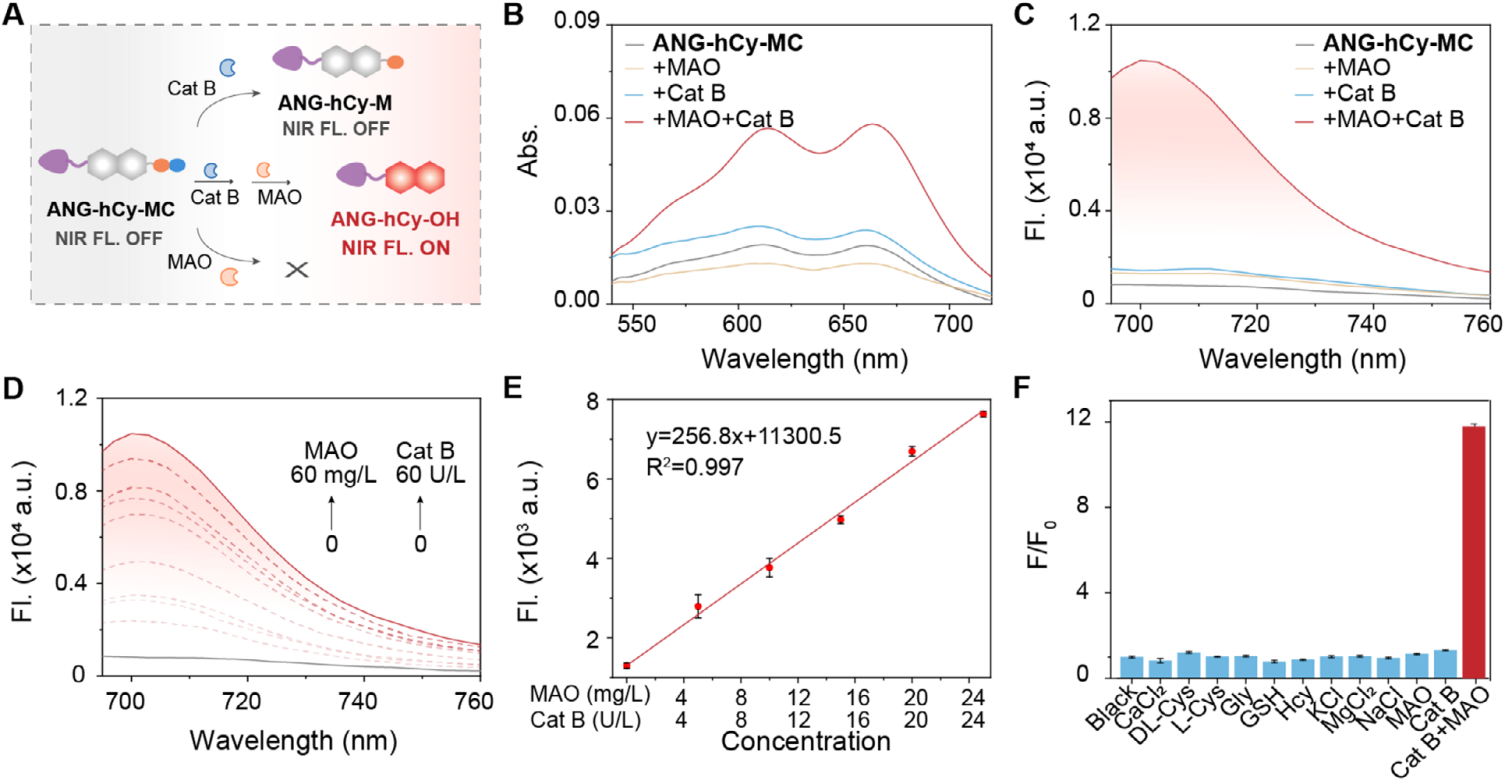
In vitro characterization of ANG‐hCy‐MC. (A) Schematic of tandem enzymatic activation mechanism. (B) UV–vis absorption and (C) fluorescence spectra of ANG‐hCy‐MC (10 µm) under four conditions: ANG‐hCy‐MC only, ANG‐hCy‐MC + MAO, ANG‐hCy‐MC + Cat B, and ANG‐hCy‐MC + Cat B + MAO in PBS (10×, pH 7.4) and EDTA (1 mm) at 37°C for 10 h. (D) Concentration‐dependent fluorescence response of ANG‐hCy‐MC (10 µm) in the presence of different concentrations of Cat B (0–60 U/L) and MAO (0–60 mg/L). (E) Linear calibration curves at low enzyme concentrations: Cat B (0–26 U/L) and MAO (0–26 mg/L). (F) Selectivity assessment: Fluorescence intensity at 708 nm after incubating ANG‐hCy‐MC with biologically relevant analytes. *λ*_ex/em_ = 680/708 nm.

Following successful structural confirmation, we systematically evaluated the photochemical properties of ANG‐hCy‐MC under various enzymatic conditions to validate its proposed dual‐enzyme cascade responsive mechanism (Figure 1A). The ultraviolet‐visible (UV–vis) absorption and fluorescence emission profiles of the probe were examined under four distinct conditions: probe alone, with MAO only, with Cat B only, and with both Cat B and MAO. Notably, only in the presence of both Cat B and MAO did ANG‐hCy‐MC exhibit a significant increase in absorption intensity (Figure 1B) and a strong fluorescence turn‐on at 708 nm (Figure 1C), confirming the essential role of the sequential two‐enzyme cascade in activating the probe. Minimal signal changes were observed upon incubation with either enzyme alone, underscoring the necessity of cooperative enzymatic cleavage for fluorescence activation. The fluorescence intensity at 708 nm showed a clear concentration‐dependent enhancement with increasing levels of both MAO and Cat B (Figure 1D). There is a good linear relationship between fluorescence intensity and enzyme concentration: y = 256.8 x + 11300.5 (R^2^ = 0.997), demonstrating the quantitative potential of the probe for sensing enzymatic activity (Figure 1E). Furthermore, ANG‐hCy‐MC displayed high specificity toward the target enzymes, as no significant fluorescence signal was triggered by various biologically relevant interfering substances (Figure 1F), highlighting its excellent selectivity in complex environments. Subsequently, ANG‐hCy‐MC exhibited excellent light stability, as both the probe itself and its activated product maintained stable fluorescence signals under continuous excitation (Figure S3).

### 2.3 Validation of the Dual‐Enzyme Cascade Activation Mechanism of hCy‐MC

**FIGURE 2:**
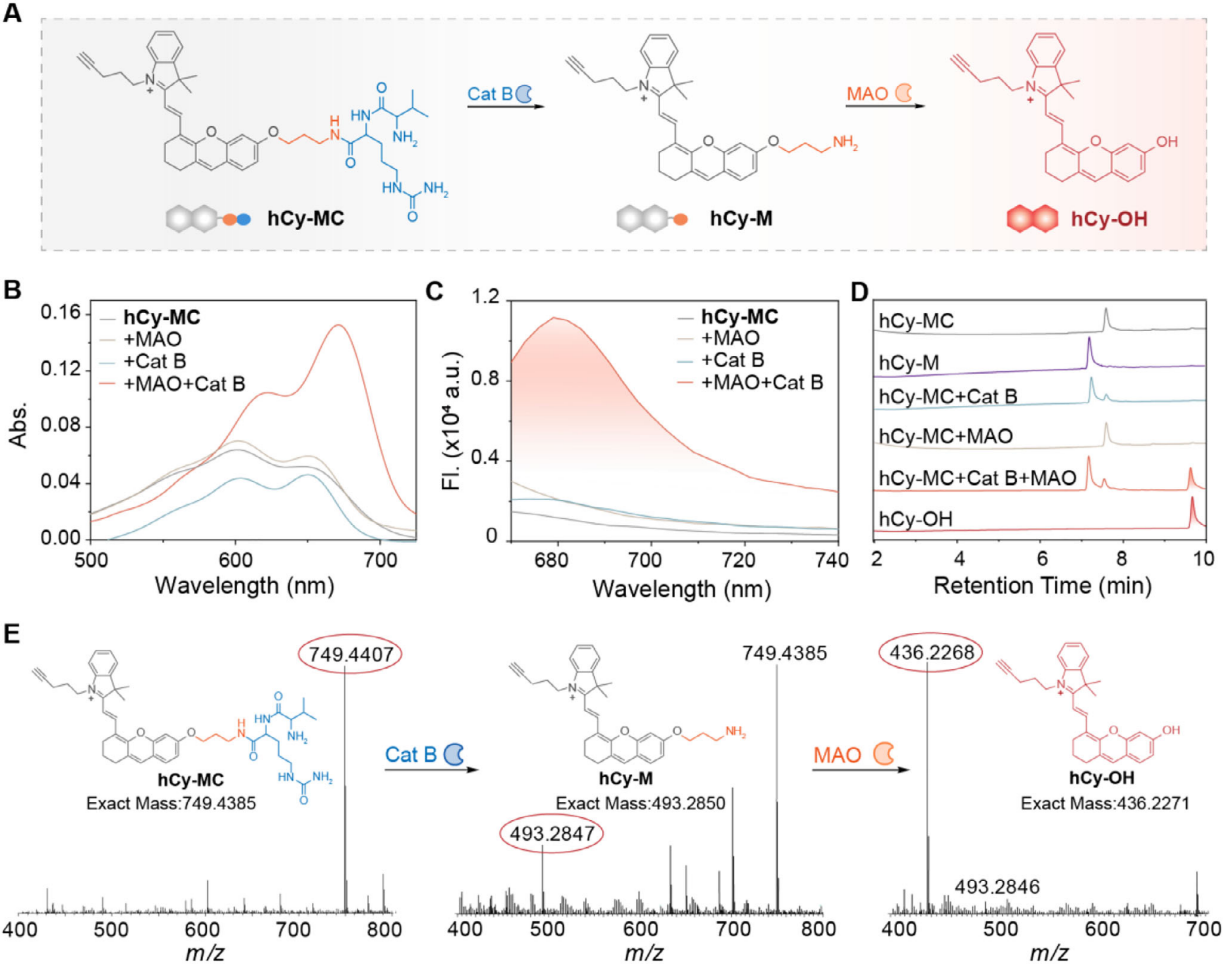
In vitro characterization of validation of the cascade activation mechanism of hCy‐MC. (A) Schematic diagram of the sequential activation of hCy‐MC against Cat B and MAO. (B) UV–vis absorption and (C) fluorescence spectra of ANG‐hCy‐MC (10 µm) under four conditions: hCy‐MC only, hCy‐MC + MAO, hCy‐MC + Cat B, and hCy‐MC + Cat B + MAO in PBS (10×, pH 7.4) and EDTA (1 mm) at 37°C for 10 h. (D) Chromatograms verifying the response mechanism of hCy‐MC. (E) HRMS validation: hCy‐MC, hCy‐MC + MAO, and hCy‐MC + MAO + Cat B. *λ*_ex/em_ = 660/680 nm.

A clear understanding of the activation mechanism is essential for developing enzymatically controlled molecular probes. To elucidate the sequential activation process of hCy‐MC (Figure 2A), we conducted a comprehensive investigation using spectroscopy, HPLC and HRMS. Characterization under conditions identical to ANG‐hCy‐MC confirmed that hCy‐MC exhibits significant UV–vis absorption enhancement and fluorescence amplification at 680 nm only upon co‐incubation with Cat B and MAO (Figure 2B,C). The distinct emission maximum of hCy‐MC (680 nm) differs from that of ANG‐hCy‐MC (708 nm), indicating that ANG conjugation influences the electronic environment of the fluorophore. Concentration‐dependent studies showed a linear increase in fluorescence intensity with enzyme concentration (Figure S4A). Moreover, the probe exhibited high specificity, with negligible activation in the presence of various biological interferents (Figure S4B).

To validate the sequential activation mechanism, in which Cat B first hydrolyzes the amide bond, followed by MAO‐mediated oxidative deamination to release the fluorescent hCy‐OH, we performed HPLC and HRMS analyses (Figure 2A). Incubation of hCy‐MC (retention time: 7.8 min, m/z = 749.4407 [M]^+^) with Cat B yielded the intermediate hCy‐M (retention time: 6.8 min, m/z = 493.2847 [M]^+^; Figure 2D,E). MAO alone did not alter the retention time of hCy‐MC, demonstrating that the Cat B‐cleavable segment acts as an essential gatekeeper preventing premature activation by MAO. Sequential treatment with both enzymes produced a new chromatographic peak corresponding to hCy‐OH (retention time: 9.7 min, m/z = 436.2268 [M]^+^), confirming complete conversion to the final fluorescent product. These results provide multi‐faceted evidence for the cascade activation mechanism wherein sequential enzymatic processing is required for fluorescence emission. This gating strategy minimizes off‐target activation and establishes a robust foundation for high‐fidelity molecular imaging in complex biological environments.

### 2.4 Highly Specific Imaging of GBM Cells With ANG‐hCy‐MC

**FIGURE 3:**
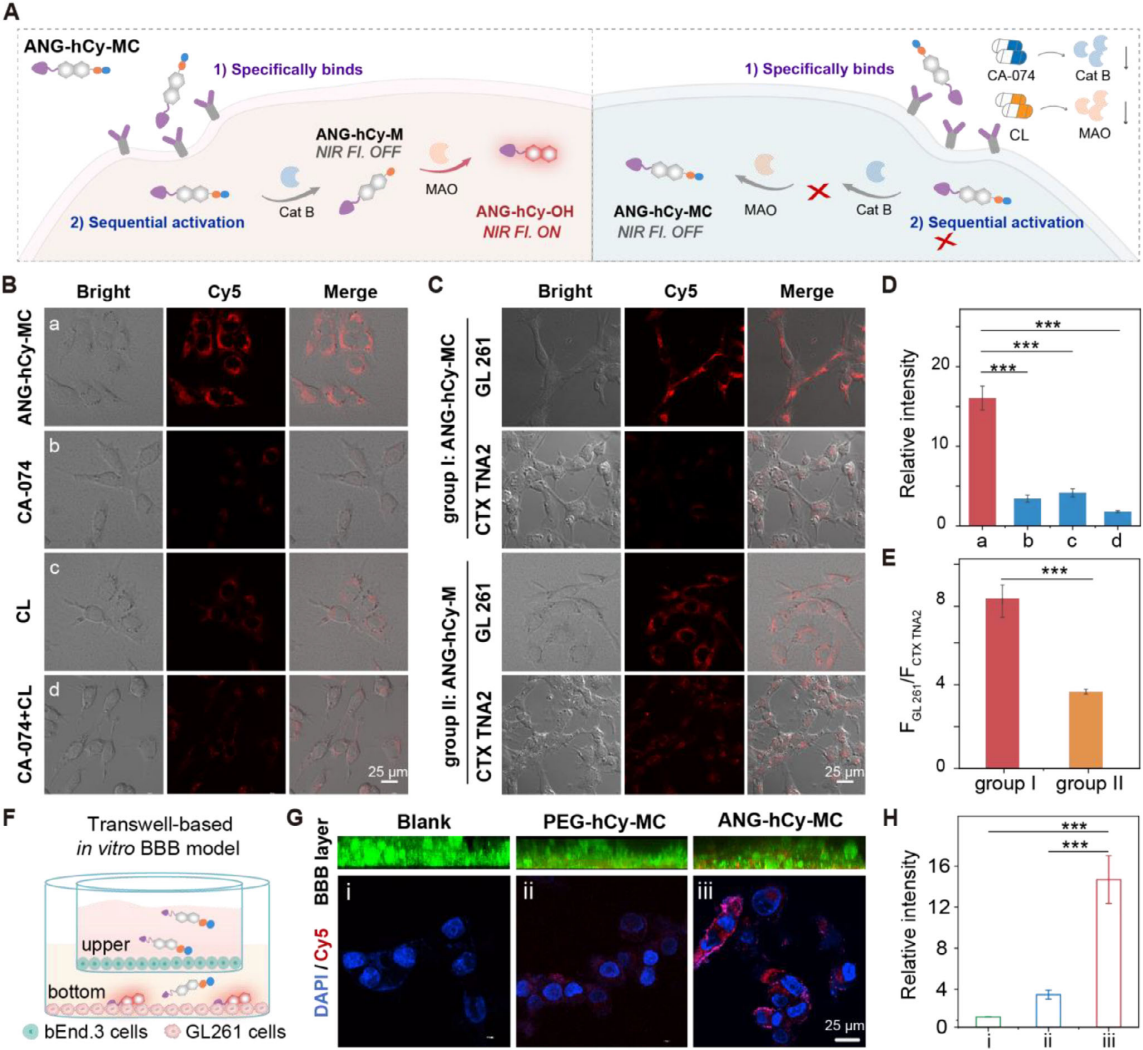
Cellular fluorescence imaging of ANG‐hCy‐MC in GL261 GBM cells and in vitro evaluation of BBB penetration. (A) Schematic of probe activation in GBM cells. (B) Confocal imaging of GL261 cells incubated with ANG‐hCy‐MC (10 µm) under enzyme inhibition conditions: (a) Untreated control; (b) Pre‐treated with Cat B inhibitor CA‐074 (100 µm, 2 h); (c) Pre‐treated with MAO inhibitor clorgyline (CL, 100 µm, 2 h); (d) Co‐treated with CA‐074 + CL. (C) Comparative imaging in GL261 (glioma) versus CTX TNA2 (normal) astrocytes. (D) Quantitative analysis of fluorescence intensity in (B). (E) Fluorescence intensity ratio in GL261 and CTX TNA2 cells (F_GL261_/F_CTX TNA2_) in (C). ^***^ *p* < 0.001. Scale bar: 25 µm. (F) Schematic illustration of the transwell‐based in vitro BBB model. (G) Representative CLSM images of GL261 cells in the lower chamber (bottom row): (i) untreated control (Blank); (ii) PEG‐hCy‐MC‐treated and (iii) ANG‐hCy‐MC‐treated groups. Scale bar: 25 µm. (H) Quantitative analysis of fluorescence intensity in GL261 cells from (G). Data are presented as means ± standard deviation (SD), n = 3, ^***^ *p* < 0.001.

In order to evaluate the ANG‐hCy‐MC targeting of glioma living cells and the sequential enzymatic activation process, we utilized GL261 and U87 glioma cell lines, both confirmed to express Cat B and MAO [39, 40, 41]. Cytotoxicity assays demonstrated the good biocompatibility of the probe in the relevant cell lines (Figures S5 and S6). As shown in Figure 3A, ANG‐hCy‐MC mediates the targeted entry of LRP1 into glioma cells GL261 (U87) [42]. Subsequently, we used specific inhibitors to evaluate the relationship between the activation of the probe in GL261 (U87) cells and the intracellular enzyme activity: using CA‐074 to inhibit Cat B, and using clogegin (CL) to inhibit MAO [43, 44]. Strong fluorescence was observed in untreated cells incubated with ANG‐hCy‐MC, indicating efficient activation by endogenous enzymes. In contrast, pretreatment with either inhibitor markedly reduced fluorescence, confirming the essential roles of both Cat B and MAO in probe activation (Figure 3B). Quantitative analysis established a strong correlation between fluorescence intensity and enzymatic activity levels. As anticipated, pronounced fluorescence activation of ANG‐hCy‐MC occurred exclusively in GL261 cells exhibiting sufficient concomitant activity of both endogenous MAO and Cat B. Similarly, as shown in Figure S9, the cells treated with the inhibitor were co‐incubated with ANG‐hCy‐M. When only CA‐074 pre‐treated the cells, the probe still showed fluorescence in GL261 (U87), indicating that ANG‐hCy‐M is only activated by MAO enzymes. Collectively, these cellular imaging results demonstrate that the designed ANG‐hCy‐MC probe functions as intended, exhibiting the characteristic cascade activation mechanism that requires the presence of both target enzymes.

To further evaluate tumor‐specific recognition capability of ANG‐hCy‐MC, CTX TNA2 astrocytes (normal cells with low enzymatic activity) served as a comparative model [45]. Parallel experiments were conducted with the MAO‐specific probe ANG‐hCy‐M (Figure S7A–C). Comparative imaging in GL261 (glioma) and CTX TNA2 (normal) cells revealed distinct activation patterns: ANG‐hCy‐MC produced markedly stronger signals in GL261 versus CTX TNA2 cells, whereas ANG‐hCy‐M elicited substantial off‐target activation in normal cells (Figure 3C). Quantitative evaluation demonstrated a significantly higher fluorescence intensity ratio (GL261/ CTX TNA2) for ANG‐hCy‐MC compared to ANG‐hCy‐M (^***^ *p* < 0.001; Figure 3E), highlighting the superior specificity afforded by its dual‐enzyme cascade activation mechanism. This gain in specificity arises because ANG‐hCy‐MC requires concurrent expression of both Cat B and MAO, where conditions met in glioma cells but not in normal astrocytes. In contrast, ANG‐hCy‐M, activated solely by MAO, produces signals even in normal cells due to basal MAO expression, resulting in lower discrimination capability. Consistent results were obtained in U87 glioma cells (Figure S10), confirming the broad applicability of ANG‐hCy‐MC across GBM subtypes and underscoring its robustness against tumor heterogeneity. In addition, four cell lines were selected for validation: U87 (human glioma cells), GL261 (mouse glioma cells), CTX TNA2 (normal glial cells), and HL‐7702 (normal liver cells). As shown in Figure S11A, incubation with the dual lock probe ANG‐hCy‐MC led to a marked increase in fluorescence intensity in tumor cells, whereas almost no fluorescence was detected in the normal cell lines. In contrast, after treatment with the single lock probe ANG‐hCy‐M, although tumor cell fluorescence was elevated, considerable fluorescence was still observed in normal cells. Quantitative analysis of fluorescence (Figure S11B) revealed that the two probes produced comparable signals in glioma cells. Notably, in the two normal cell lines, the fluorescence intensity of the single lock probe ANG‐hCy‐M was 2–3 fold higher than that of the dual lock probe ANG‐hCy‐MC. In summary, the cascade activation design of ANG‐hCy‐MC represents a substantial advance in molecular imaging by mitigating off‐target activation, thus enabling high‐specificity visualization of glioma cells.

Next, we used an in vitro transmembrane model to demonstrate the effective BBB permeability of ANG‐hCy‐MC. Previous studies have confirmed that ANG, a ligand of LRP1, facilitates molecular transport across the BBB via receptor‐mediated endocytosis. To assess the active BBB‐traversing capacity conferred by ANG, we employed a transwell‐based in vitro BBB model, with the non‐targeted probe PEG‐hCy‐MC as a control. As illustrated in Figure 3F, the transwell model was established by seeding with bEnd.3 cells in the upper chamber to form a monolayer mimicking the BBB, while GL261 glioma cells were seeded in the lower chamber. Immunofluorescence staining of the tight junction protein ZO‐1 showed continuous and well‐organized intercellular junctions (Figure S12), confirming the integrity and functionality of the endothelial barrier. Before permeability assays, we verified that PEG‐hCy‐MC retained the dual‐enzyme cascade activation behavior, responding only to concurrent Cat B and MAO stimulation, thereby validating its use as an appropriate non‐targeting control (Figure S8A–C). Following 48‐h incubation with either ANG‐hCy‐MC or PEG‐hCy‐MC in the upper chamber, Confocal laser scanning microscope (CLSM) imaging of GL261 cells in the lower chamber was conducted to evaluate probe penetration and subsequent activation. The confocal imaging images of the upper petri dish are shown in Figure S13. CLSM images clearly revealed efficient traversal of the bEnd.3 endothelial barrier by ANG‐hCy‐MC, followed by its internalization and red fluorescence activation within GL261 cells (Figure 3G). In contrast, PEG‐hCy‐MC showed markedly limited barrier penetration and minimal fluorescence signal in the target cells. Quantitative analysis confirmed that the fluorescence intensity in the ANG‐hCy‐MC group was over 3‐fold higher than that in the PEG‐hCy‐MC group, a difference that was statistically significant (Figure 3H). These findings conclusively demonstrate that ANG conjugation significantly enhances the BBB permeability of ANG‐hCy‐MC, enabling efficient accumulation and activation in glioma cells. This high translocation efficiency provides a solid experimental basis for further in vivo application and underscores the potential of ANG‐hCy‐MC in targeting GBM.

### 2.5 In Vivo Targeted Fluorescence Enrichment Verification of ANG‐hCy‐MC in an Orthotopic GL261 GBM Mouse Model

Initial evaluation in subcutaneous xenografts demonstrated that intratumoral pretreatment with either Cat B inhibitor CA‐074 or MAO inhibitor CL significantly reduced fluorescence signals compared to the PBS control group (Figure S14A‐C), supporting the enzyme‐dependent activation mechanism of ANG‐hCy‐MC. From the organ dissection diagram, compared with the group treated with the inhibitory agent, this probe showed more specific activation at the tumor sites in the untreated group, and it was cleared from the liver and kidneys as expected [46] (Figure S14D). These results confirm that the dual‐enzyme cascade response is essential for high tumor‐specific signal amplification.

**FIGURE 4:**
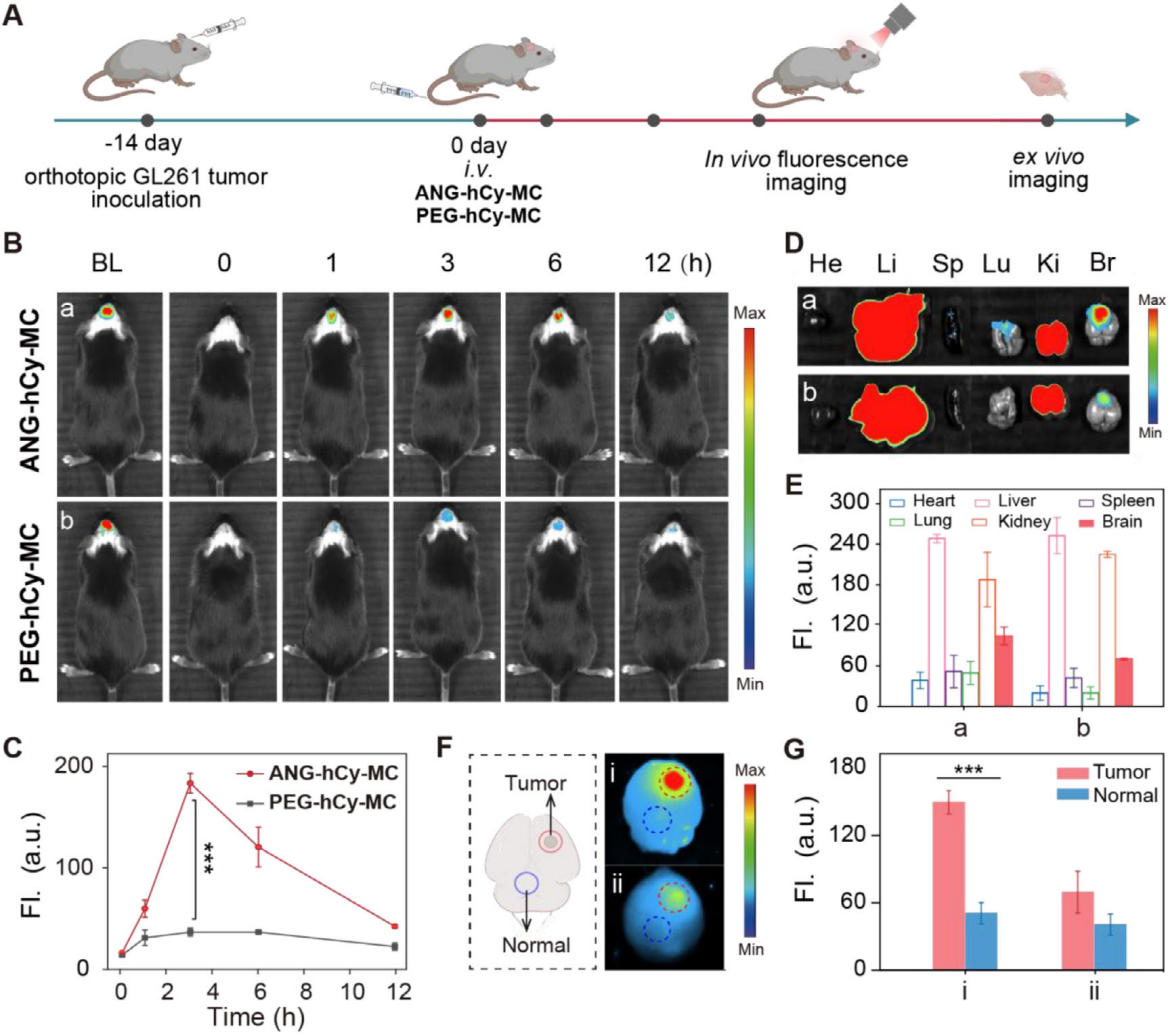
In vivo targeted fluorescence enrichment verification of ANG‐hCy‐MC in an orthotopic GL261 GBM mouse model. (A) Experimental timeline of tumor implantation and imaging sessions. (B) In vivo fluorescence imaging of tumor‐bearing mice administered with (a) ANG‐hCy‐MC and (b) PEG‐hCy‐MC. (C) Quantitative analysis of in vivo tumor fluorescence intensity in (B) (^***^ *p* < 0.001). (D) Ex vivo fluorescence images of dissected organs at 12 h post‐injection: (a) ANG‐hCy‐MC and (b) PEG‐hCy‐MC. (E) Quantification of ex vivo tumor fluorescence intensity in (D). (F) Representative brains illustrating tumor‐to‐normal contrast: (i) ANG‐hCy‐MC and (ii) PEG‐hCy‐MC (red dashed circle: tumor regions). (G) Quantification of tumor‐to‐normal brain fluorescence ratio in (F) (Data are presented as means ± standard deviation (SD), n = 3, ^***^ *p* < 0.001).

After a diagnosis of GBM, surgical removal of the tumor area remains the key to effective treatment and prognosis. Therefore, accurately delineating the tumor boundary and the micro‐invasive area is of utmost importance. The long‐term imaging of fluorescent probes provides surgeons with sufficient time to perform complex surgeries, enabling them to locate the tumor in real time and clearly display its boundary. This helps them make precise decisions during the surgery and minimizes damage to healthy brain tissue to the greatest extent. We next employed an orthotopic GL261‐Luc model to rigorously evaluate the brain‐targeting capacity and activation specificity of ANG‐hCy‐MC. Non‐invasive bioluminescence imaging confirmed tumor growth and defined the region of interest. Following intravenous administration of ANG‐hCy‐MC or non‐targeted PEG‐hCy‐MC, optical imaging was captured at various time points (Figure 4A). As shown in Figure 1, 4 after intravenous injection of the probe, the ANG‐hCy‐MC group already exhibited strong fluorescence in the GBM area, confirming the successful achievement of brain tumor targeting and the subsequent activation of the probe. Meanwhile, time‐dependent imaging showed that the fluorescence intensity at the tumor site had reached its peak 3 h after injection, and even at 12 h, the fluorescence intensity was still significant. It is worth noting that the fluorescence intensity in the glioma area of the ANG‐hCy‐MC group was stronger than that of the PEG‐hCy‐MC group. This might be due to the relatively weaker effect of PEG‐hCy‐MC in penetrating the blood‐brain barrier, or it could be the result of passive accumulation through the enhanced permeability and retention (EPR) effect. Moreover, the background signal in non‐tumor brain regions was extremely low, highlighting its high specificity. This might be attributed to the dual‐enzyme cascade activation strategy, which has significant advantages as it can reduce background signals and achieve specific recognition of GBM. However, the control probe PEG‐hCy‐MC showed weak fluorescence at the tumor site after injection, and the fluorescence intensity at the 3h time point was 6‐fold lower than that of ANG‐hCy‐MC (Figure 4C). This undoubtedly proves that the active targeting mediated by ANG and the trans‐basement membrane transport mediated by LRP1 have crucial advantages over the passive drug delivery mechanism.

After 12 h of fluorescence imaging, the organs and brains of the mice were dissected for in vitro imaging (Figure 4D,E). The fluorescence in the GBM regions treated with ANG‐hCy‐MC was significantly higher than that in normal brain tissue. The fluorescence ratio of the tumor region to normal brain tissue (T/N) reached 4‐fold (Figure 4F,G), and the probe was mainly cleared through the liver and kidneys. In contrast, the fluorescence ratio of the tumor to normal brain tissue (T/N) in the PEG‐hCy‐MC group was only about 1.7‐fold. Therefore, PEG‐hCy‐MC is not suitable for precise tumor imaging and fluorescence navigation surgical resection. These results collectively indicate that the superiority of the probe is attributed to its “sequential locking and ANG modification” design. The modification of ANG gives it a stronger penetration ability through the blood‐brain barrier, which is a key characteristic not possessed by the PEG‐based control probe. Importantly, the sequential locking design enables ANG‐hCy‐MC to clearly display the tumor edge, which provides excellent contrast for guiding surgical resection. Collectively, this figure validates the probe's excellent BBB permeability, and tumor‐specific accumulation, which are the prerequisites for precise intraoperative guidance. In addition, the hemolysis analysis and hematoxylin and eosin (H&E) staining revealed no significant physiological abnormalities across all treatment groups, supporting the biosafety profile of ANG‐hCy‐MC and PEG‐hCy‐MC for in vivo imaging (Figures S15 and S16).

### 2.6 Fluorescence Navigation Surgical Resection of GBM and Cell‐Level Boundary Delineation

**FIGURE 5:**
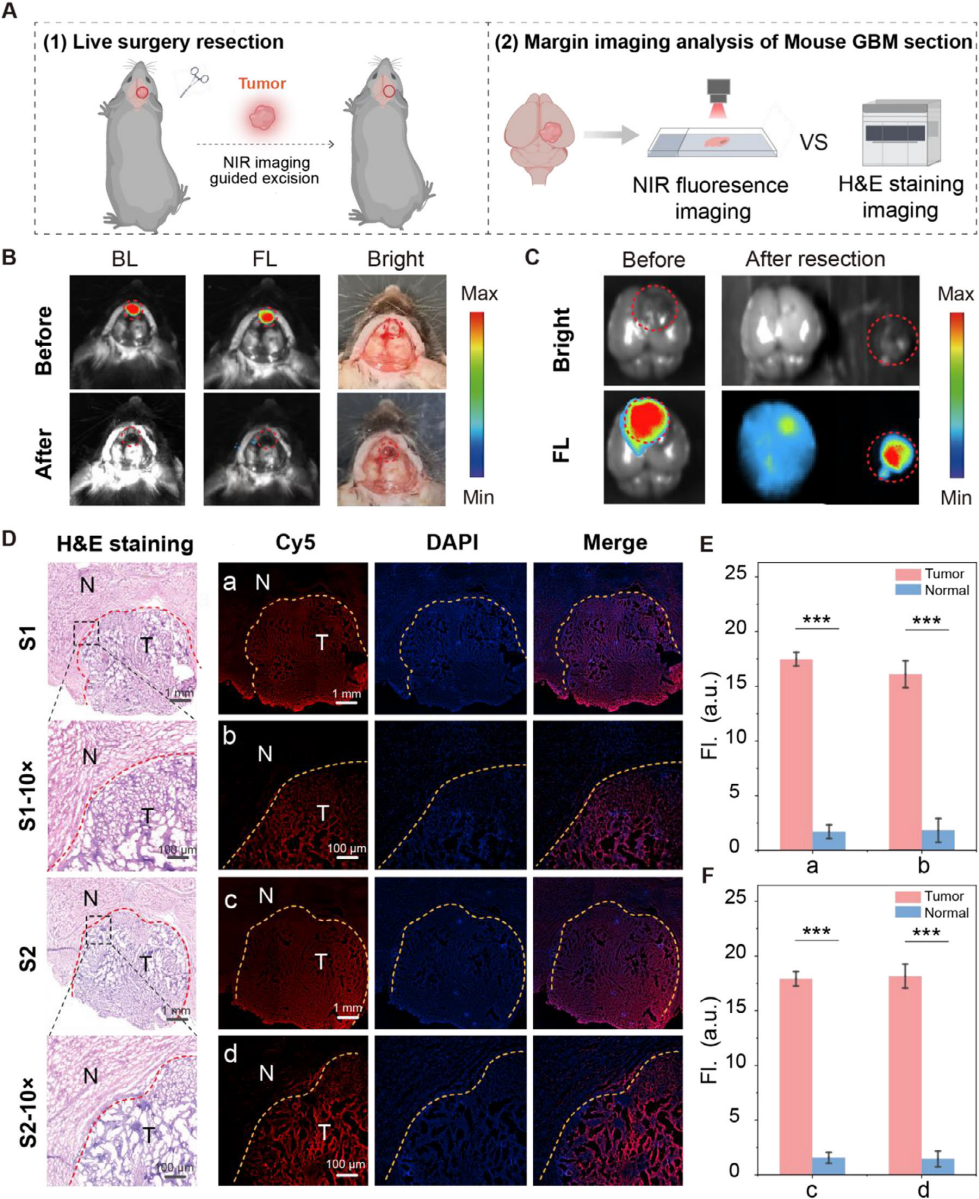
Fluorescence navigation tumor resection and cellular‐level margin delineation in orthotopic GBM models. (A) Schematic illustrating the procedure for fluorescence navigation tumor resection, and margin assessment in orthotopic models. (B) Intraoperative bioluminescence and fluorescence images before and after tumor resection (the red dashed area represents the GBM and the resection area). (C) Ex vivo brain image confirming complete tumor resection. *λ*_ex/em_ = 680/710 nm. (D) Margin analysis in murine GBM: H&E staining and corresponding ANG‐hCy‐MC fluorescence (10 µm, 30 min) at (a,c) low and (b,d) high magnifications. Scale bars: (a,c) 1 mm; (b,d) 100 µm. Dotted lines indicate tumor margins. (E) Quantification of fluorescence intensity in tumor versus adjacent normal regions in (D(a,b)). (F) Quantification of fluorescence intensity in tumor versus adjacent normal regions in (D(c,d)). (Data are presented as means ± standard deviation (SD), n = 3, ^***^ *p* < 0.001.).

Benefitting from the outstanding tumor‐targeting capability and high imaging contrast of ANG‐hCy‐MC in GBM, we further explored its potential for guiding the surgical resection in an orthotopic GL261 model. GBM often infiltrates into the adjacent healthy brain tissues, posing a significant challenge for surgeons to achieve maximal tumor removal while preserving neurological function [47]. Twelve hours after probe injection, we performed tumor resection under NIR fluorescence guidance (Figure 5A). As shown in Figure 5B, the red dashed lines delineate the GBM region. The co‐localization of bioluminescence and fluorescence signals confirms the precise targeting capability of the probe toward GBM. Under fluorescence guidance, the tumor was meticulously resected. Postoperatively, the fluorescence signal at the resection site was substantially diminished. Subsequent bioluminescence imaging further verified the complete removal of the tumor, demonstrating the efficacy of the fluorescence‐guided resection and the utility of bioluminescence as an independent validation modality. This outcome was further validated by ex vivo analysis of the resected tissue (Figure 5C), underscoring the reliability of ANG‐hCy‐MC for intraoperative guidance. Thus, this probe not only enables accurate intraoperative diagnosis and precise margin definition in orthotopic GBM models but also supports fluorescence navigation tumor resection, highlighting its translational potential for NIR image‐guided surgical applications.

Capitalizing on the high targeting specificity and activation selectivity of ANG‐hCy‐MC demonstrated in orthotopic GBM models, we further assessed its ability to delineate invasive tumor margins at cellular resolution using ex vivo tissue sections. To ensure comparability, we first characterized the glioma samples from orthotopic mouse models. Tissue cryosections (5 µm thickness) were prepared for correlative histopathological and fluorescence imaging (Figure 5A). In sections of orthotopic GL261 mouse brains, the H&E staining pathologically confirmed the tumor boundaries (Figure 5D). Fluorescence confocal microscopy revealed strong and tumor‐specific fluorescence signal from ANG‐hCy‐MC, which sharply decreased at the pathological margins, allowing clear margin discrimination even at low magnification (Figure 5D). To compare the dual‐lock probe ANG‐hCy‐MC with its single‐lock counterpart ANG‐hCy‐M, tissue fluorescence analysis was performed. As shown in Figure S17A,B, ANG‐hCy‐MC exhibited distinct fluorescence that closely aligned with the tumor border, with minimal signal in adjacent normal tissues. In contrast, ANG‐hCy‐M produced detectable fluorescence in normal tissues, and the tumor‐associated signal appeared more diffuse with less defined margins. Quantitative evaluation of the fluorescence intensity ratio between tumor and normal tissue (F_T_/F_N_) further revealed that the F_T_/F_N_ ratio for ANG‐hCy‐MC was approximately 1.5‐fold higher than that for ANG‐hCy‐M (Figure S17C). Additionally, as shown in Figure S18, distinct differences in fluorescence intensity were observed among tumor tissue, marginal regions, and normal tissue in mouse GBM sections. Quantitative fluorescence analysis demonstrated a maximum tumor‐to‐normal (T/N) ratio of 11.61‐fold (Figure 5E,F), which remained as high as 10.45‐fold even at tenfold magnification. These findings transition from demonstrating diagnostic capability to illustrating therapeutic utility, proving that ANG‐hCy‐MC is not only a sensitive imaging agent but also a powerful intraoperative tool that can guide surgeons to achieve maximal safe resection, thereby addressing the central challenge in GBM management.

### 2.7 Cellular‐Level Delineation of Invasive Margins in Human GBM Specimens

**FIGURE 6:**
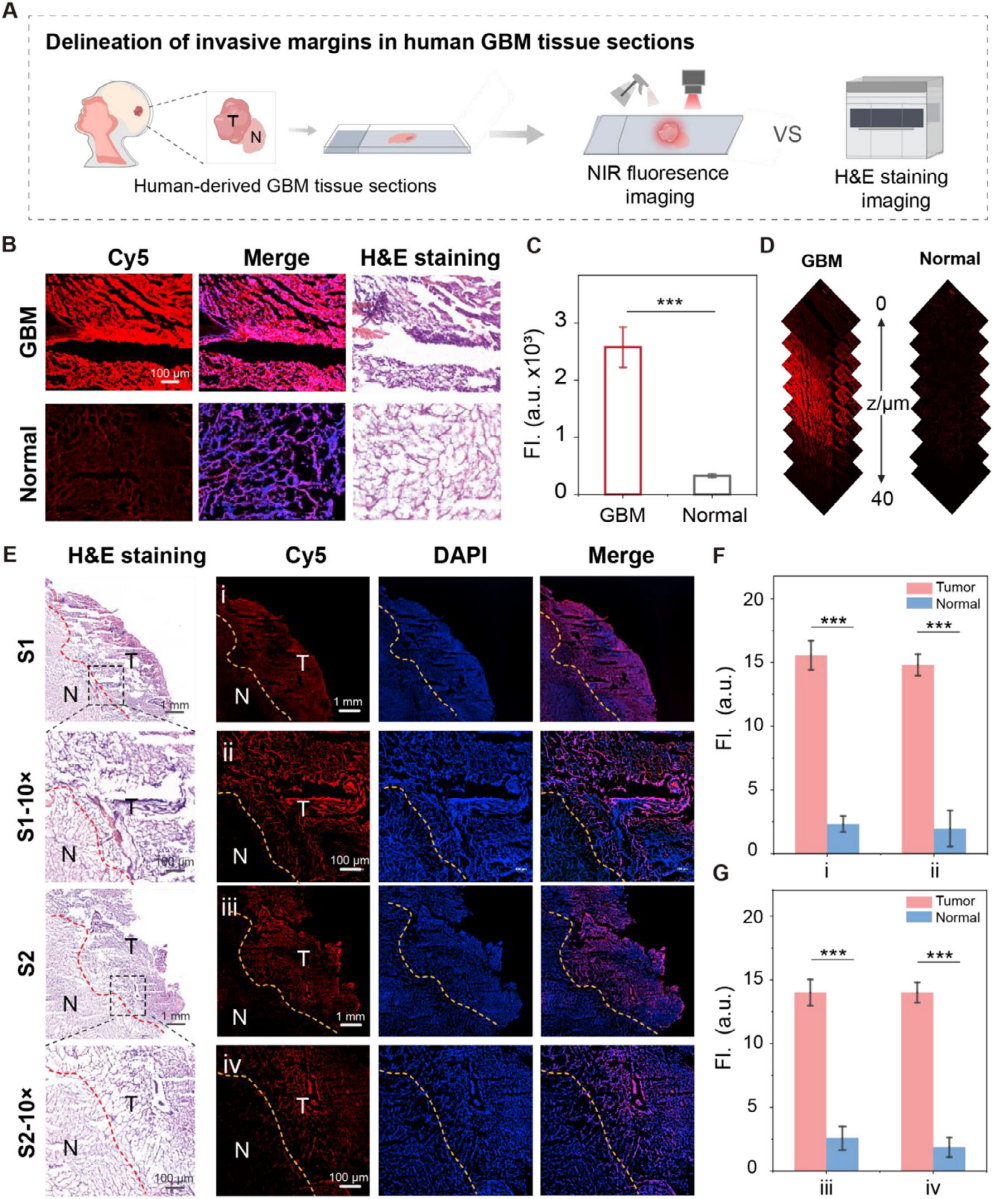
Precision delineation of invasive margins in human GBM specimens using ANG‐hCy‐MC. (A) Workflow for processing clinical GBM specimens. (B) Validation of human GBM margins: architecture defined by H&E staining and co‐localization with ANG‐hCy‐MC fluorescence (Scale bar:100 µm). (C) Fluorescence intensity in tumor foci versus normal parenchyma is shown in (B). (D) Z‐axis scanning with ANG‐hCy‐MC (10 µm) confirms 3D‐restricted activation in GBM specimens. (E) Margin analysis in clinical GBM frozen sections: H&E staining and corresponding ANG‐hCy‐MC fluorescence (10 µm, 30 min) at (i, ii) low and (iii, iv) high magnifications. Scale bars: (i, ii) 1 mm; (iii, iv) 100 µm. Dotted lines indicate GBM margin regions. (F) Quantification of fluorescence intensity in tumor versus adjacent normal regions in (E(i, ii)). (G) Quantification of fluorescence intensity in tumor versus adjacent normal regions in (E(iii, iv)). Data are presented as means ± standard deviation (SD), n = 3, ^***^ *p* < 0.001.

Based on the high targeting specificity and superior capacity for tumor boundary delineation demonstrated by ANG‐hCy‐MC in orthotopic GBM mouse models, we further evaluated its performance in delineating invasive tumor margins at the cellular level in clinical human tissue sections. All human specimens were acquired in strict accordance with approved ethical guidelines and consisted of freshly resected GBM tissues (Institutional Review Board Approval No: HDFY‐LL‐2022‐094). Tissue cryosections were prepared for correlated histopathological and fluorescence imaging (Figure 6A). Notably, pathological assessment confirmed the diagnosis of glioblastoma in the clinical specimens. H&E staining revealed that the tumor exhibited invasive growth characteristics, with poorly defined borders between the GBM and adjacent normal brain tissues (Figure S19) [48]. Fluorescence confocal microscopy showed that ANG‐hCy‐MC strongly highlighted neoplastic regions and effectively distinguished them from adjacent non‐neoplastic areas, displaying high concordance with histopathological features (Figure 6B; Figure S20). Quantitative analysis indicated significantly elevated fluorescence intensity in tumor margins compared to normal parenchyma (^***^ *p* < 0.001, Figure 6C). 3D Z‐stack reconstructions further confirmed restricted fluorescence localization within malignant regions, demonstrating a penetration depth of approximately 40 µm in human samples (Figure 6D; Figure S21), which supports an enzyme‐activated, tumor‐specific targeting mechanism.

To evaluate boundary delineation performance, co‐registered H&E and confocal images were analyzed within infiltration zones. ANG‐hCy‐MC accurately outlined even dispersed tumor cells at infiltrative margins, with fluorescence intensity profiles showing a sharp decrease at the transition to normal tissue. As shown in Figure 6E, specimens treated with ANG‐hCy‐MC exhibited strong NIR fluorescence exclusively within tumor areas, with nearly undetectable signal in adjacent normal tissues. Quantitative analysis revealed that fluorescence intensity at the tumor edge was significantly higher than that in normal parenchyma (^***^ *p* < 0.001). Even at ten fold magnification, a T/N ratio of up to 7.83‐fold was maintained (Figure 6F,G). Although ANG‐hCy‐MC achieves excellent tumor‐to‐normal tissue contrast, the inherent heterogeneity of human GBM may lead to the emergence of signal patterns that reveal the complexity and invasiveness of the disease. Furthermore, the fluorescence‐defined boundaries showed high consistency with histopathologically confirmed margins in H&E staining. These results demonstrate that ANG‐hCy‐MC can specifically target human glioma cells and clearly delineate tumor margins, indicating substantial promise for improving surgical guidance. The high signal‐background gradient enables precise identification of invasive fronts at single‐cell resolution. These results demonstrate that ANG‐hCy‐MC achieves exceptional clarity and precision in outlining glioma infiltration boundaries in both orthotopic mouse models and human clinical specimens. Its ability to provide microscopically resolved, histologically correlated margin delineation underscores its strong potential as an intraoperative tool for guiding precision resection in glioma surgery.

## 3 Conclusions

In summary, we have successfully designed and developed a novel cascade recognition of activatable probe ANG‐hCy‐MC, for precision GBM imaging and fluorescence surgical navigation. Its design incorporates ANG functionalization for LRP1 receptor‐mediated BBB crossing and tumor‐specific accumulation, coupled with a tandem enzymatic cascade activation mechanism (Cat B/MAO) that minimizes background signal and false positives in healthy tissue by requiring sequential enzymatic cleavage for fluorescence activation. Key findings demonstrate that ANG‐hCy‐MC achieves exceptionally high tumor‐to‐normal contrast and single‐cell resolution at invasive margins in both orthotopic mouse models and human GBM specimens, enabling precise real‐time delineation of tumor boundaries with minimal background interference. The demonstrated efficacy in guiding surgical resection underscores its strong clinical translatability, establishing ANG‐hCy‐MC as a transformative platform for improving the completeness of GBM resection and enhancing patient outcomes through fluorescence surgical navigation.

## 4 Experimental Section

### 4.1 Materials

All chemicals and solvents were purchased commercially and used without further purification. ANG‐N_3_(TFFYGGSRGKRNNFKTEEYC‐PEG_45_‐N_3_) and PEG_45_‐N_3_ were purchased from Xi'an ruixi biological technology company. All cell culture media, fetal bovine serum (FBS), DMEM, antibody against ZO‐1, Cy3‐conjugated goat anti‐rabbit, goat serum, and phosphate‐buffered saline (PBS, pH 7.4) were purchased from Biosharp. The column chromatography was performed using silica gel (200‐300 mesh, Qingdao Ocean Chemicals, Qingdao, China). The ultrapure water (18 Ω) used in all experiments was obtained from the Milli‐Q system (Millipore, USA).

### 4.2 Confocal Fluorescence Imaging of Cells

For cell fluorescence imaging of ANG‐hCy‐MC or ANG‐hCy‐M, GL261 or U87 cells were plated in a glass‐bottom dish for 12 h. Cell fluorescence imaging of ANG‐hCy‐MC and ANG‐hCy‐M in GL261 or U87 cells can be divided into four groups: (a) Unprocessed GL261 or U87 cells; (b) GL261 or U87 cells were pre‐incubated with CA‐074 (100 µm) for 2 h; (c) GL261 or U87 cells were pre‐incubated with CL (100 µm) for 2 h; (d) GL261 or U87 cells were pre‐incubated with CA‐074 (100 µm) and CL (100 µm) for 2 h. Confocal fluorescence imaging of cells using ANG‐hCy‐MC or ANG‐hCy‐M (10 µm) for 4 h. Fluorescence images of the cells were captured by a Nikon AX R microscope (*λ_ex_* = 640 nm, *λ_em_* = 662–737 nm). For compare the differences in cell fluorescence imaging between ANG‐hCy‐MC and ANG‐hCy‐M, GL261 (U87) or CTX TNA2 cells were plated in a glass‐bottom dish for 12 h. Cell fluorescence imaging of ANG‐hCy‐MC and ANG‐hCy‐M in GL261 (U87) or CTX TNA2 cells can be divided into two groups: In group, GL261 (U87) and CTX TNA2 cells treated with 10 µm ANG‐hCy‐MC for 4 h. In group, GL261 (U87) and CTX TNA2 cells treated with 10 µm ANG‐hCy‐M for 4 h. Fluorescence images of the cells were captured by a Nikon AX R microscope.

### 4.3 In Vivo Imaging of the GL261 Tumor‐Bearing Mouse Model

Female C57BL/6 mouse (6–8 weeks) were purchased from Beijing Huafukang Biotechnology Co, Ltd. All animal experiments were carried out in accordance with the guidelines for Care and Use of Laboratory Animals of Beijing University of Technology, China, and approved by the Animal experiment Ethics (HS202202011). GL261 tumor‐bearing mouse were randomly divided into 3 groups (n = 3 per group) two days prior to imaging: (a) untreated control, (b) intratumoral injection of CA‐074 (15 mg/kg), and (c) intratumoral injection of CL (15 mg/kg). The probe ANG‐hCy‐MC (150 µm) was then administered intravenously, and fluorescence imaging was performed at 0, 2, 4, 6, and 10 h post‐injection. At the 10 h time point, all mice were euthanized, and major organs (heart, liver, spleen, lung, kidney) along with tumors were collected, rinsed with saline, and subjected to ex vivo fluorescence imaging. The images of the organs were taken using IVIS imaging system as described above (*λ_ex_*/*λ_em_* = 680/710 nm).

### 4.4 In Vivo Imaging of the in Orthotopic GL261 GBM Mouse Model

*GBM Mouse* were randomly divided into 2 groups (n = 3 per group): (a) Injected with ANG‐hCy‐MC (150 µm), (b) Injected with PEG‐hCy‐MC. Then, mice were performed in vivo fluorescence imaging at 0, 1, 3, 6, and12 h after injection. After the final in vivo imaging time point, mice were deeply anesthetized and subjected to intracardiac perfusion with a sufficient volume of ice‐cold saline until the effluent from the right atrium became clear. This procedure ensured thorough removal of blood and circulating probes from the vascular system. Brain and other organs were then harvested for ex vivo imaging and analysis. After completely removing the tissues containing fluorescence signals, imaging is conducted again. The images of the organs were taken using IVIS imaging system as described above (*λ_ex_* /*λ_em_* = 680/710 nm).

### 4.5 Imaging of Margins in Mouse GBM Tissue

Animal procedures were conducted in accordance with the guidelines of the Institutional Animal. Fresh tumor tissues were immediately rapidly frozen in liquid nitrogen (−80°C for storage). Subsequently, they were cut into thin slices (5 micrometers thick) using a cryostat. All the tissue samples were sectioned using the Leica CM1950 fully automatic cryostat. Sections were incubated with probe ANG‐hCy‐MC (ANG‐hCy‐M) (10 µm) for 30 mins, then imaged after washing by PBS (3×). Then, the cells were stained with DAPI staining solution (0.5 µg/mL) for 5 mins, and then rinsed 3 times with PBS. Fluorescence images of the tissue sections were captured by a Nikon AX R confocal laser scanning microscope. (*λ_ex_* = 640 nm, *λ_em_* = 662–737 nm)

### 4.6 Processing and Imaging of Clinical Human Samples

The GBM human tissue samples were collected during the GBM surgery of patients, and all patients in the study gave informed consent for their participation in the study. All human specimens were acquired in strict accordance with approved ethical guidelines and consisted of freshly resected GBM tissues (Institutional Review Board Approval No: HDFY‐LL‐2022‐094). Subsequently, tissues were snap‐frozen in liquid nitrogen immediately (stored at −80°C), the frozen tissue blocks were sectioned using a constant temperature cryostat. The temperature inside the sectioning chamber was typically maintained at −20°C to −25°C, and the section thickness was usually 5 µm, which was used for subsequent experiments. All the tissue samples were sectioned using the Leica CM1950 fully automatic cryostat. Similarly, sections were incubated with probe ANG‐hCy‐MC (10 µm) for 30 mins, then imaged after washing by PBS (3×). Then, the cells were stained with DAPI staining solution (0.5 µg/mL) for 5 mins, and then rinsed with PBS (3×). Fluorescence images of the tissue sections were captured by a Nikon AXR confocal laser scanning microscope. (*λ*_ex_ = 640 nm, *λ*_em_ = 662–737 nm)

### 4.7 Statistical Analysis

The fluorescence intensity of region of interest was analyzed by Image J. All statistical analysis were performed using Origin. The data were analyzed using Student's t‐test, and represented as mean ± standard deviation (SD). ^**^ *p* < 0.01, ^***^ *p* < 0.001 as indicated in the figure legends. For each experiment, unless otherwise noted, the data under each condition were accumulated from at least three independent experiments.

## Conflicts of Interest

The authors declare no conflicts of interest.

## Supporting information




**Supporting File**: advs74331‐sup‐0001‐SuppMat.docx

## Data Availability

The data that support the findings of this study are available from the corresponding author upon reasonable request.
