# Supplementary material for: A Cascade Recognition of Activatable Probe for Fluorescence Navigation Glioblastoma Surgery: Overcoming Blood‐Brain Barrier and Off‐Target Limitations
Source: Adv Sci (Weinh). 2026 Feb 10;13(22):e21404. doi: 10.1002/advs.202521404 (PMC13088282; doi:10.1002/advs.202521404)
Supplement: Supplementary file 1 — Supporting File: advs74331‐sup‐0001‐SuppMat.docx [file ADVS-13-e21404-s001.docx]

**Supporting** **Information**

A Cascade Recognition of Activatable Probe for Fluorescence Navigation Glioblastoma Surgery: Overcoming Blood-Brain Barrier and Off-Target Limitations

Xinru An^#^, Yu Guo^#^, Yongning Bian, Yanli Tan, Chuan Fang, Hengzhu Zhang, Xueqian Chen, Lin Yuan^*^, Dongdong Su^*^

**Table of Contents**

1. Instruments.........................................................................................................................S2

2. Synthesis and Characterizations ........................................................................................S2

3. General Methods.................................................................................................................S4

4. Supporting Figures..............................................................................................................S6

5. NMR and MS Spectra.........................................................................................................S15

**1. Instruments**

Spectroscopic data was measured on a FS5 spectrofluorometer (Edinburgh Instruments Ltd., UK). The UV-vis absorption spectrum of all probes was acquired by a spectrophotometer (Shimadzu UV-1800, Japan). High performance liquid chromatography (HPLC) was performed on Agilent Infinity 1260 with a C18 column (100 Å, 30 × 4.6 mm). Test conditions: detection wavelength: 600 nm, mobile phase: acetonitrile/H_2_O (0.1% CF_3_COOH) = 5/95 (*v/v*) to 95/5 (*v/v*), flow rate: 1 mL/min. High-resolution electrospray ionization mass spectrometry (HRMS-ESI) was performed using an LTQ Orbitrap XL mass spectrometer (Thermo Scientific, USA). ^1^H NMR and ^13^C NMR spectra were recorded on a Bruker Advance spectrometer with TMS as an internal standard. Cell images were acquired by a Nikon confocal laser scanning microscope (Nikon AXR microscope). The *in vivo* fluorescence images of mouse were acquired by an IVIS spectrum system (PerkinElmer, USA). The fluorescence intensity of region of interest was analyzed by Living Image 4.5 Software.

**2. Synthesis and Characterizations**

***Synthesis of hCy-OH***

hCy-OH were prepared by the reported methods^[1]^. ^1^H NMR (400 MHz, DMSO-*d*_6_) δ8.48 (d, *J* = 14.4 Hz, 1H), 7.70 (d, *J* = 7.4 Hz, 1H), 7.59 – 7.55 (m, 2H), 7.47 (d, *J* = 9.8 Hz, 2H), 7.36 (t, *J* = 7.3 Hz, 1H), 6.87 (s, 1H), 6.40 (d, *J* = 14.4 Hz, 1H), 4.34 (t, *J* = 6.8 Hz, 2H), 3.01 (s, 1H), 2.68 (d, *J* = 29.8 Hz, 4H), 2.25 (s, 2H), 1.95 (s, 1H), 1.83 (s, 2H), 1.74 (s, 6H). ^13^C NMR (151 MHz, DMSO-*d*_6_) δ175.19, 161.46, 155.38, 142.25, 141.84, 136.37, 129.89, 129.14, 126.29, 124.64, 123.11, 117.35, 115.14, 114.80, 112.35, 102.53, 102.12, 83.92, 72.75, 50.07, 41.79, 28.19, 26.90, 24.77, 20.61, 15.78. HR-MS C_30_H_30_NO_2_^+^ (ESI, *m/z*): calculate (M)^+^: 436.2271, found (M)^+^: 436.2278.

***Synthesis of hCy-M***

hCy-OH (436 mg, 1 mmol) was mixed with DMF. Next, tert-butyl N- (3-bromopropyl) carbamate (544 mg, 2 mmol) and K_2_CO_3_ (179 mg, 1.3 mmol) were added slowly under a nitrogen atmosphere and the reaction was carried out overnight at 50 °C. Then, the mixture was diluted with ethyl acetate (30 mL) and washed three times with water (40 mL). The organic layer was separated and dried over anhydrous Na_2_SO_4_. The solvent was removed by evaporation under reduced pressure and the residue was subjected to silica gel chromatography (DCM: MeOH = 25: 1) as eluent to give a purple-blue solid hCy-MBoc (328 mg, 57%). To a solution of hCy-MBoc (300 mg, 1 mmol) in anhydrous CH_2_Cl_2_ (5 mL) at room temperature, added the mixed solution of trifluoroacetic acid (0.5 mL) and anhydrous CH_2_Cl_2_ (2 mL). The resulting mixture was stirred a room temperature for 1 h, then the solvent was removed by evaporation under reduced pressure. The residue was washed using diethyl ether to give a blue solid hCy-M was a purple solid (289 mg, 95% yield). ^1^H NMR (400 MHz, DMSO-*d*_6_) δ 8.61 (d, *J* = 14.8 Hz, 1H), 7.75 (d, *J* = 7.4 Hz, 1H), 7.69 (d, *J* = 8.0 Hz, 1H), 7.56 (t, *J* = 5.7 Hz, 2H), 7.47 (d, *J* = 7.4 Hz, 1H), 7.10 (s, 1H), 7.02 (d, *J* = 10.7 Hz, 1H), 6.57 (d, *J* = 14.9 Hz, 1H), 4.44 (t, *J* = 7.3 Hz, 2H), 4.15 (t, *J* = 5.9 Hz, 2H), 3.13 (d, *J* = 6.2 Hz, 2H), 3.01 (s, 1H), 2.71 (d, *J* = 30.7 Hz, 4H), 2.26 (s, 2H), 1.99 (s, 2H), 1.89 (s, 2H), 1.85 (s, 1H), 1.78 (s, 6H). ^13^C NMR (151 MHz, DMSO-*d*_6_) δ177.95, 162.43, 161.32, 156.13, 154.36, 145.45, 142.53, 141.89, 134.18, 129.31, 127.46, 123.20, 115.94, 114.63, 113.38, 104.49, 101.99, 83.85, 78.02, 72.85, 50.87, 41.79, 32.70, 28.73, 28.04, 26.90, 24.77, 22.91, 20.39, 15.77. HR-MS C_33_H_37_N_2_O_2_^+^ (ESI, *m/z*): calculated (M)^+^: 493.2850, found (M)^+^: 493.2862.

***Synthesis of hCy-MC***

hCy-M (250 mg, 1 mmol) was mixed with CH_2_Cl_2_. Next, Boc-Val-Cit-OH (195 mg, 1.2 mmol), (1-Ethyl-3-(3-dimethyllaminopropyl) carbodiimide hydrochloride) EDC (15.7 mg, 1.1 mmol) and (1-Hydroxybenzotriazole) HOBT (71.9 mg, 1.1 mmol) were added slowly, and DIPEA (0.5 mmol) was dropped and the reaction was carried out overnight at room temperature. Then, the mixture was diluted with CH_2_Cl_2_ (40 mL) and washed three times with water (40 mL). The organic layer was separated and dried over anhydrous Na_2_SO_4_. The solvent was removed by evaporation at low pressure and the residue was subjected to silica gel chromatography (DCM: MeOH = 22: 1) as eluent to give a blue solid (120 mg, 49%). To a solution of hCy-MCBoc (100 mg, 1mmol) in anhydrous CH_2_Cl_2_ (5 mL) at room temperature, added the mixed solution of trifluoroacetic acid (0.5 mL) and anhydrous CH_2_Cl_2_ (2 mL). The resulting mixture was stirred a room temperature for 1 h, then the solvent was removed by evaporation under reduced pressure. The residue was washed using diethyl ether to give a blue solid hCy-MC was a purple-blue solid (92 mg, 89%). ^1^H NMR (400 MHz, Methanol-*d*_4_) δ 8.78 (d, *J* = 14.7 Hz, 1H), 7.65 (d, *J* = 7.3 Hz, 1H), 7.56 (d, *J* = 8.0 Hz, 1H), 7.48 (d, *J* = 8.2 Hz, 1H), 7.43 (s, 2H), 7.06 (s, 1H), 6.99 (d, *J* = 8.2 Hz, 1H), 6.57 (d, *J* = 14.7 Hz, 1H), 4.45 (t, *J* = 7.4 Hz, 3H), 4.18 (d, *J* = 6.0 Hz, 2H), 3.73 (d, *J* = 5.6 Hz, 1H), 3.14 (s, 1H), 2.76 (d, *J* = 18.7 Hz, 4H), 2.40 (s, 2H), 2.19 (s, 1H), 2.05 (d, *J* = 11.7 Hz, 4H), 1.93 (s, 2H), 1.83 (s, 6H), 1.04 – 0.99 (m, 6H). ^13^C NMR (101 MHz, Methanol-*d*_4_) δ 177.77, 162.69,162.05,154.53, 142.03, 134.25, 128.76, 127.18, 126.89, 122.42, 115.91, 114.62, 113.55, 112.23, 108.71, 103.14,101.34, 100.93, 82.30, 70.28, 66.08, 58.16, 50.57,44.16, 43.51, 35.87, 30.20, 28.92, 28.64, 27.07, 26.09, 23.97, 20.17, 17.55, 16.51, 15.13. HR-MS C_44_H_57_N_6_O_5_^+^ (ESI, *m/z*): calculated (M)^+^: 749.4385, found (M)^+^: 749.4407.

**Scheme S1:** Synthesis route of the hCy-M and hCy-MC.

***Synthesis of ANG-hCy-MC or ANG-hCy-M***

hCy-MC (12 mg, 1 mmol) and ANG-N_3_ (78 mg, 1 mmol) were dissolved in DMF. Then, CuBr (5.7 mg, 0.5 mmol) and sodium ascorbate (2.8 mg, 0.8 mmol) in DMF were added. The reaction mixture was stirred at room temperature under N_2_ atmosphere for 24 h. Then the reaction solution was dialyzed against Deionized water for 24 h to remove salts and DMF. Pure ANG-hCy-MC was obtained after freeze drying and determined by ^1^H-NMR spectroscopy. The ANG-hCy-M were prepared under the same synthetic procedure except that the hCy-MC was replaced with hCy-M.

***Synthesis of PEG-hCy-MC***

hCy-MC (12 mg, 1 mmol) and PEG45-N3 (32 mg, 1 mmol) were dissolved in DMF. Then, CuBr (5.7 mg, 0.5 mmol) and sodium ascorbate (2.8 mg, 0.8 mmol) in DMF were added. The reaction mixture was stirred at room temperature under N2 atmosphere for 24 h. Then the reaction solution was dialyzed against Deionized water for 24 h to remove salts and DMF. Pure PEG-hCy-MC was obtained after freeze drying and determined by ^1^H-NMR spectroscopy.

**Scheme S2:** Synthesis routes of the ANG-hCy-MC, ANG-hCy-M and PEG-hCy-MC.

**3.General Methods**

***Cell culture and in vitro cytotoxicity assay***

GL261 cells (mouse glioma cells), U87 cells (human glioma cells), bEnd.3 cells (mouse brain-derived endothelial cells.3) , CTX TNA2 cells (normal glial cells) and HL-7702 (normal liver cells) were cultured in cultured in Dulbecco's modified eagle's medium (DMEM), supplemented with 10% fetal bovine serum (FBS) and 1% penicillin (100 U/mL)/streptomycin (100 U/mL). To evaluate the in vitro safety of ANG-hCy-MC and ANG-hCy-M, CCK-8 assay was performed in GL261 cell lines. Cells (1×10^5^ cells per well) were seeded into 96-well plates and incubated at 37°C for overnight. Then the culture medium was replaced by fresh medium containing samples at various concentrations, and the cells were further incubated for overnight. The cells were treated with ANG-hCy-MC and ANG-hCy-M at the desired hCy concentrations. then, 10% CCK-8 solution was added to each well, the absorbance was finally measured at 450 nm by a microplate reader.

***BBB penetration studies in vitro***

In order to study the transcytosis efficiency of ANG-hCy-MC, an in vitro blood−brain barrier (BBB) model was constructed using a 6-well transwell cell culture system. The specific experimental process is as follows: the experimental steps included placing the Transwell insert in the well plate, loading bEnd.3 cells (1 × 10^5^ cells/well) onto the Transwell membrane insert, and inoculating GL261 cells (5 × 10^4^ cells/well) at the bottom. After 24h incubation period, when the bEnd.3 cells grew into a dense cell layer. First, rinse the monolayer cells of bEnd.3 with PBS solution, fix the cells at room temperature with 4% paraformaldehyde solution for 15 mins, and then thoroughly wash with PBS to remove paraformaldehyde. Nonspecific binding was minimized by incubating the cells with a blocking solution consisting of 10% goat serum in PBS for 1 hour. Subsequently, the primary antibody against ZO-1 was applied at a dilution of 1:1000 and allowed to interact with the cells overnight at 4 °C. After the primary antibody incubation, the cells were again washed with PBS to remove any unbound antibody. Cy3-conjugated goat anti-rabbit secondary antibody, diluted 1:200 in PBS, was then added and incubated with the cells at room temperature for 30 mins to facilitate signal detection (Cy3: *λ_em_*=550-610nm). Then, bEnd. 3 cells were placed in DAPI staining solution (0.5 μg/mL) for 5 mins before rinsing with PBS. Immunofluorescence analysis of the tight junction protein ZO-1 was performed using a confocal laser scanning microscope to evaluate the integrity of the bEnd.3 cell monolayer. The intact bEnd.3 cell monolayer effectively blocked the substance exchange between the upper and lower chambers of the Transwell device, thereby simulating the BBB function in vitro. Fluorescence images of the cells were captured by a Nikon AX R microscope. After the construction of the BBB model in vitro, the probes ANG-hCy-MC (10 μM) and PEG-hCy-MC (10 μM) were added to the upper chamber of the in vitro BBB model. After 48 h of incubation, the GL261 cells were treated with 4% paraformaldehyde solution for 15 mins, and then washed 3 times with PBS. GL261 cells were subjected to DAPI staining solution (0.5 μg/mL) for 5 mins. The Z axis scan of ANG-hCy-MC in bEnd.3 cells and the uptake of ANG-hCy-MC and PEG-hCy-MC in GL261 cells were analyzed by confocal laser scanning microscope. Fluorescence images of the lower layer cells were captured by a Nikon AX R microscope (*λ_ex_*=640 nm, *λ_em_*=662-737 nm).

***The GL261 tumor-bearing mouse model***

Female C57BL/6 mouse (6–8 weeks) were purchased from Beijing Huafukang Biotechnology Co., Ltd. All animal experiments were carried out in accordance with the guidelines for Care and Use of Laboratory Animals of Beijing University of Technology, China and approved by the Animal experiment Ethics (HS202202011). First, GL261 cells (1×10^5^) were subcutaneously inoculated into the lateral thigh area of hind legs of C57BL/6 female mice. When the size of the tumor reaches 5 mm × 5 mm, subsequent experiments can be conducted.

***Orthotopic GL261 GBM mouse model***

Female C57BL/6 mouse (6-8 weeks) anesthetized with Zoletil and xylazine were fixed with brain stereotactic instruments. After disinfection with povidone-iodine, the scalp was cut, and the anterior fontanelle was exposed. The injection point was localized 0.5 mm anterior and 2.0 mm right lateral to the bregma. Then, a hole was carefully made with a skull drill, and the microinjector was inserted vertically to 3.5 mm depth and retracted to 3 mm. Next, 3 μL of GL261-luc cell suspension (2 × 10^5^ cells) was injected into the localized brain area. After 5 mins of retention, the needle was slowly withdrawn for 1 min. Finally, the burr hole was sealed with sterile bone wax, and scalp sutured with absorbable sutures and about two weeks later, the tumor formed.

***Live surgery resection***

Fluorescence surgery-guided resection treatment was performed on the mice. Twelve hours after the probe injection, the mice were deeply anesthetized and the skin of each mouse was dissected using scissors to fully expose the skull. Then, forceps were used to separate the skull and the mouse brain tissue was exposed to remove the GBM, bioluminescence imaging and fluorescence imaging were conducted both before and after the surgery.

***Extracorporeal surgical resection***

After the probe injection, the mice were deeply anesthetized 12 hours later, and a sufficient amount of frozen physiological saline was injected into their hearts for perfusion until the fluid flowing out of the right atrium became clear. This operation ensured that the blood and the circulating probe in the vascular system were completely removed. Subsequently, the brain was collected to remove the GBM, and fluorescence imaging was performed before and after the surgery.

***Histology***

For the validation biosecurity, healthy mice were treated with ANG-hCy-MC and PEG-hCy-MC for 48 h, then the mouse were sacrificed. The major organs (heart, liver kidney, spleen, lung) were fixed in 4 % formaldehyde solution and embedded in paraffin. Then, the tissue samples were sectioned and stained by hematoxylin and eosin (H&E). All the photographs were recorded on a Nikon ECLIPSE Ti2-U inverted fluorescence microscope equipped with an IXON-L-888 cameras (ANDOR, UK).

1. **Supporting Figures**


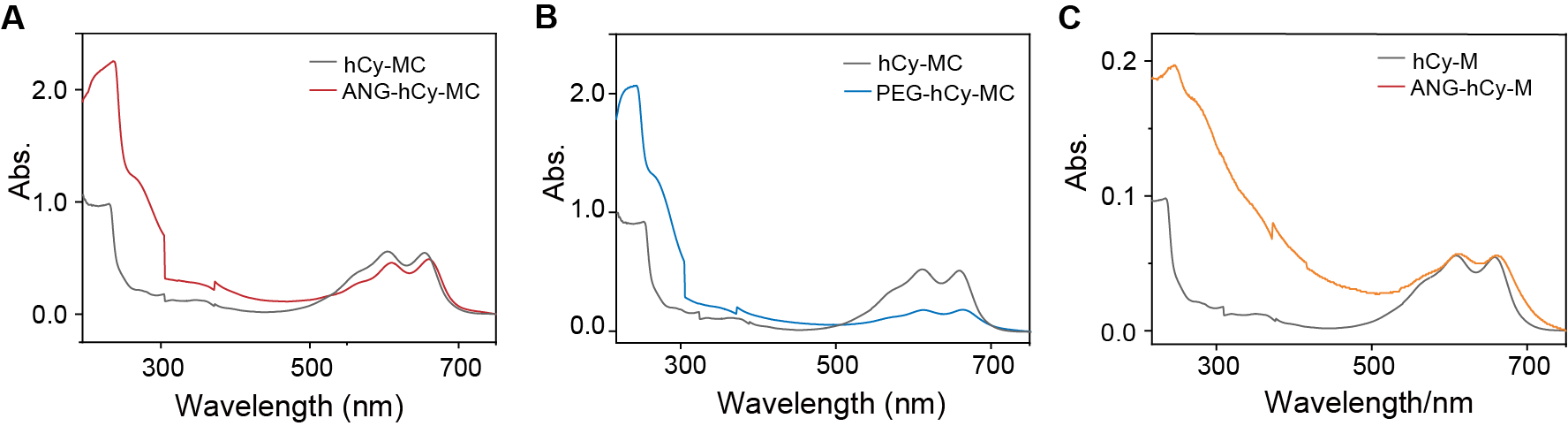


**Figure S1:** (A) UV-vis absorption of hCy-MC and ANG-hCy-MC.(B) hCy-MC and PEG-hCy-MC. (C) hCy-M and ANG-hCy-M.


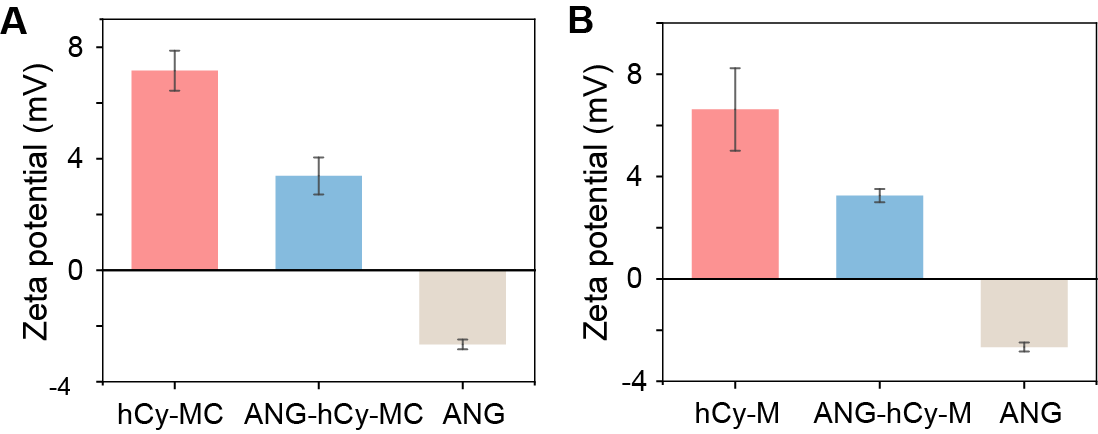


**Figure S2:** (A) Zeta potentials of hCy-MC, ANG-hCy-MC and ANG, (B) Zeta potentials of hCy-M, ANG-hCy-M and ANG.


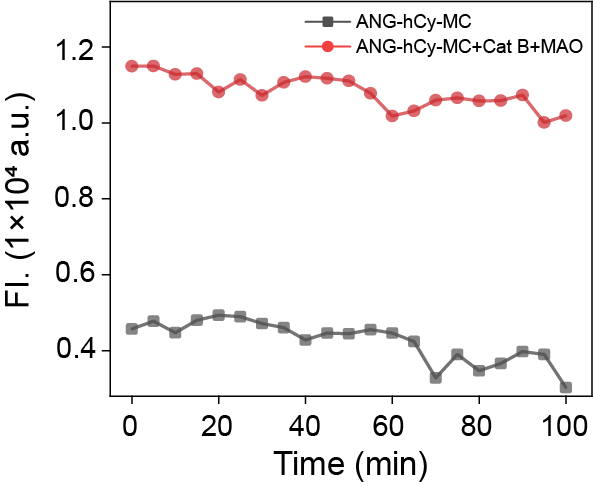


**Figure S3：**The photostability of ANG-hCy-MC and ANG-hCy-MC+ Cat B+ MAO within 100 mins.


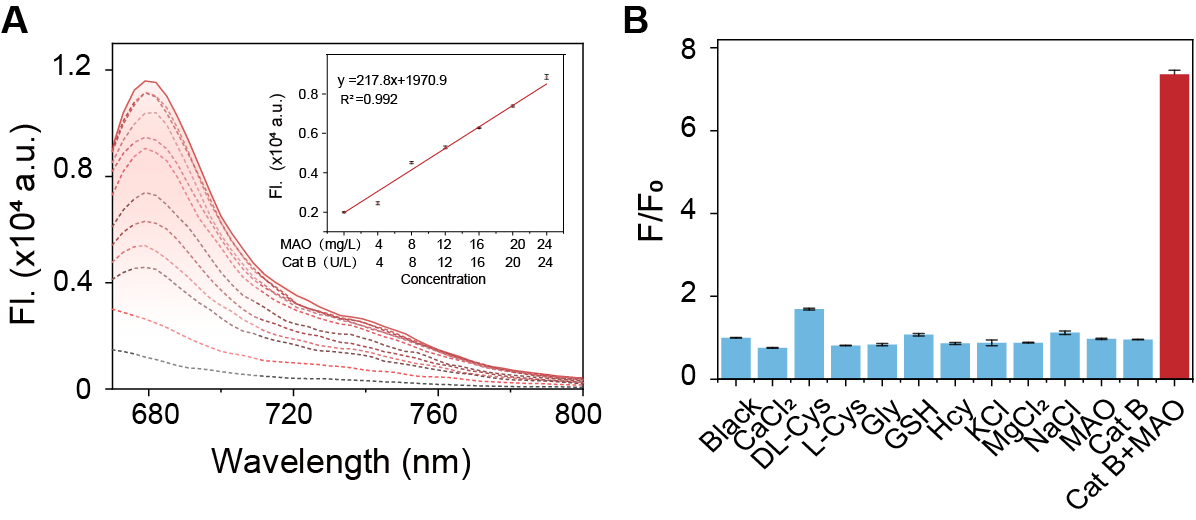


**Figure S4:** (A) Fluorescence spectra of hCy-MC (10 μM) in the presence of different concentrations of Cat B and MAO; Linear fluorescence response at low concentrations of Cat B and MAO. (B) Fluorescence intensity at 708 nm after incubation of hCy-MC with various analytes. *λ_ex_* /*λ_em_*= 660/680 nm.


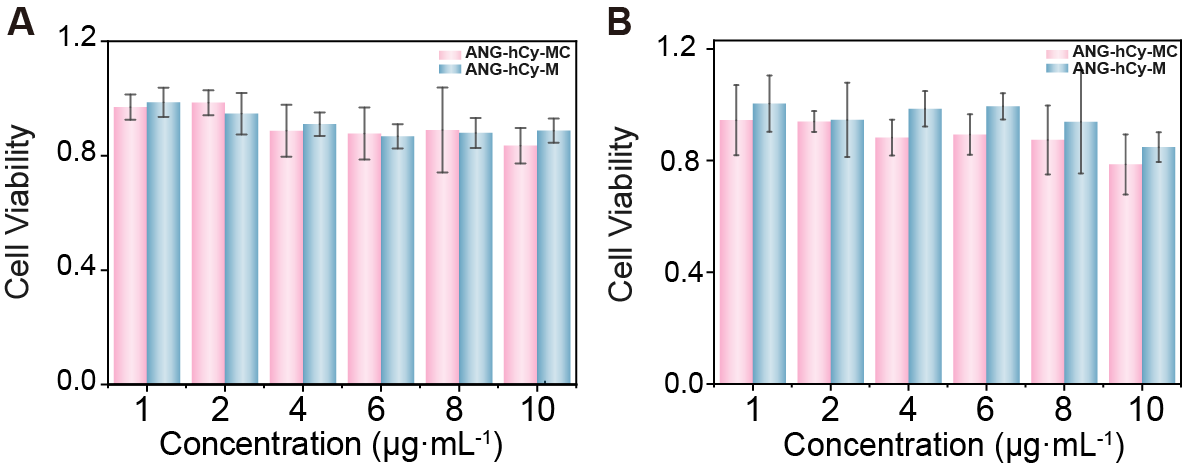


**Figure S5:** The cytotoxicity of ANG-hCy-MC and ANG-hCy-M in (A) GL261 cells and (B) U87 cells.


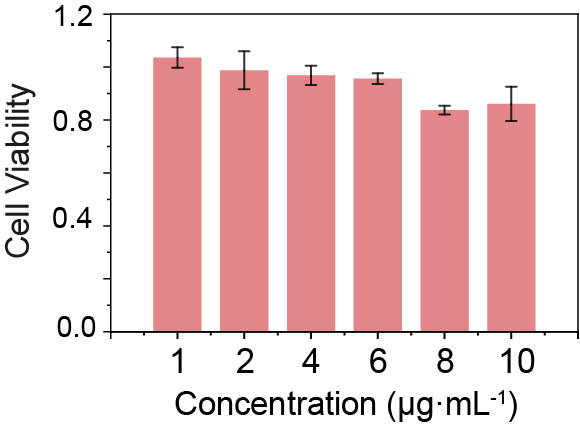


**Figure S6:** The cytotoxicity of PEG-hCy-MC in GL261 cells.


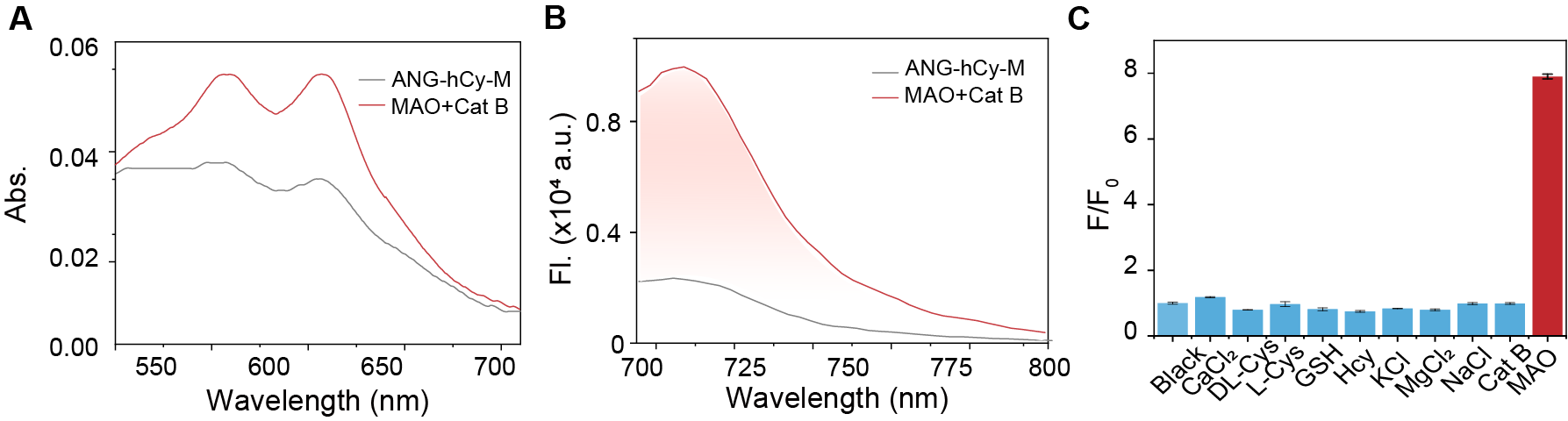


**Figure S7:** (A) UV-vis absorption and (B) fluorescence spectra of ANG-hCy-M (10 μM) under the conditions of ANG-hCy-M and ANG-hCy-M with MAO (60 mg/L) in PBS (10×, pH 7.4) at 37 °C for 10 h. (C) Fluorescence intensity at 708 nm after incubation of ANG-hCy-M with various analytes. λ_ex_ /λ_em_= 680/708 nm.


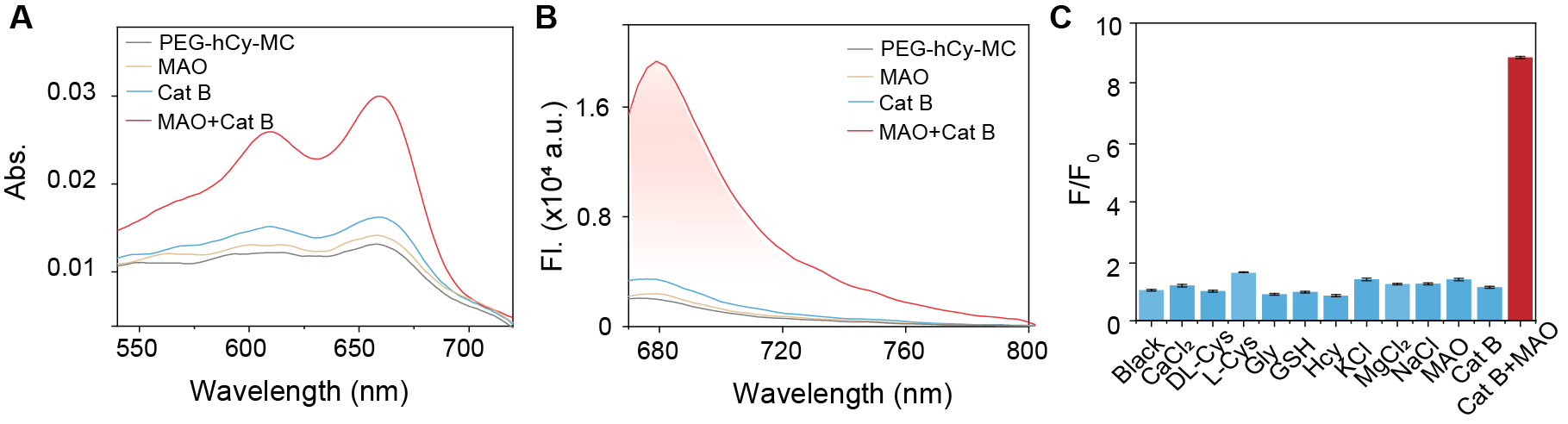


**Figure S8:** (A) UV-vis absorption and (C) fluorescence spectra of PEG-hCy-MC (10 μM) under the four conditions of PEG-hCy-MC alone, PEG-hCy-MC + MAO, PEG-hCy-MC + Cat B and PEG-hCy-MC + Cat B and MAO in PBS (10×, pH 7.4) and EDTA (1 mM) at 37 °C for 10 h. *λ_ex_* /*λ_em_*= 660/680 nm.


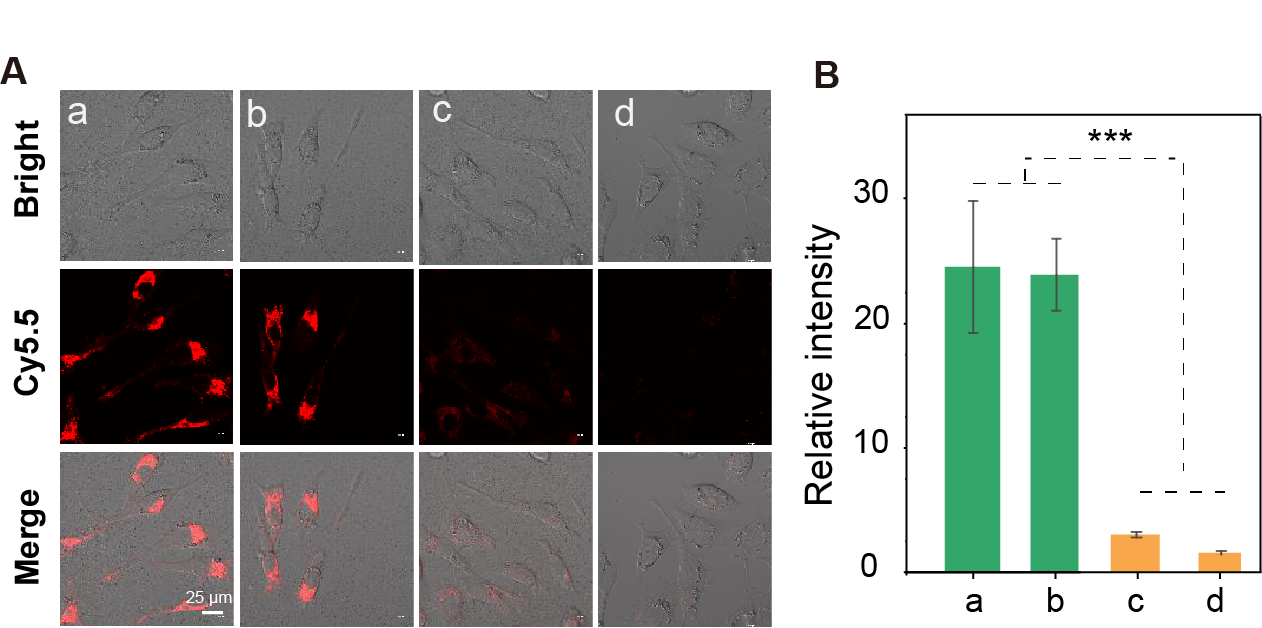


**Figure S9:** Fluorescence imaging of ANG-hCy-M in GL261 cells**.** (A) Confocal fluorescence imaging of GL261 cells using ANG-hCy-M (10 µM). (a) Unprocessed GL261 cells; (b) GL261 cells were pre-incubated with CA-074 (100 µM) for 2 h; (c) GL261 cells were pre-incubated with CL (100 µM) for 2 h; (d) GL261 cells were pre-incubated with CA-074(100 µM) and CL (100 µM) for 2 h. *λ_ex_*=640 nm, *λ_em_*=662-737 nm. Scale bar: 25 µm. (Data are expressed as means ± standard deviation (SD), n = 3, ***P < 0.001.)


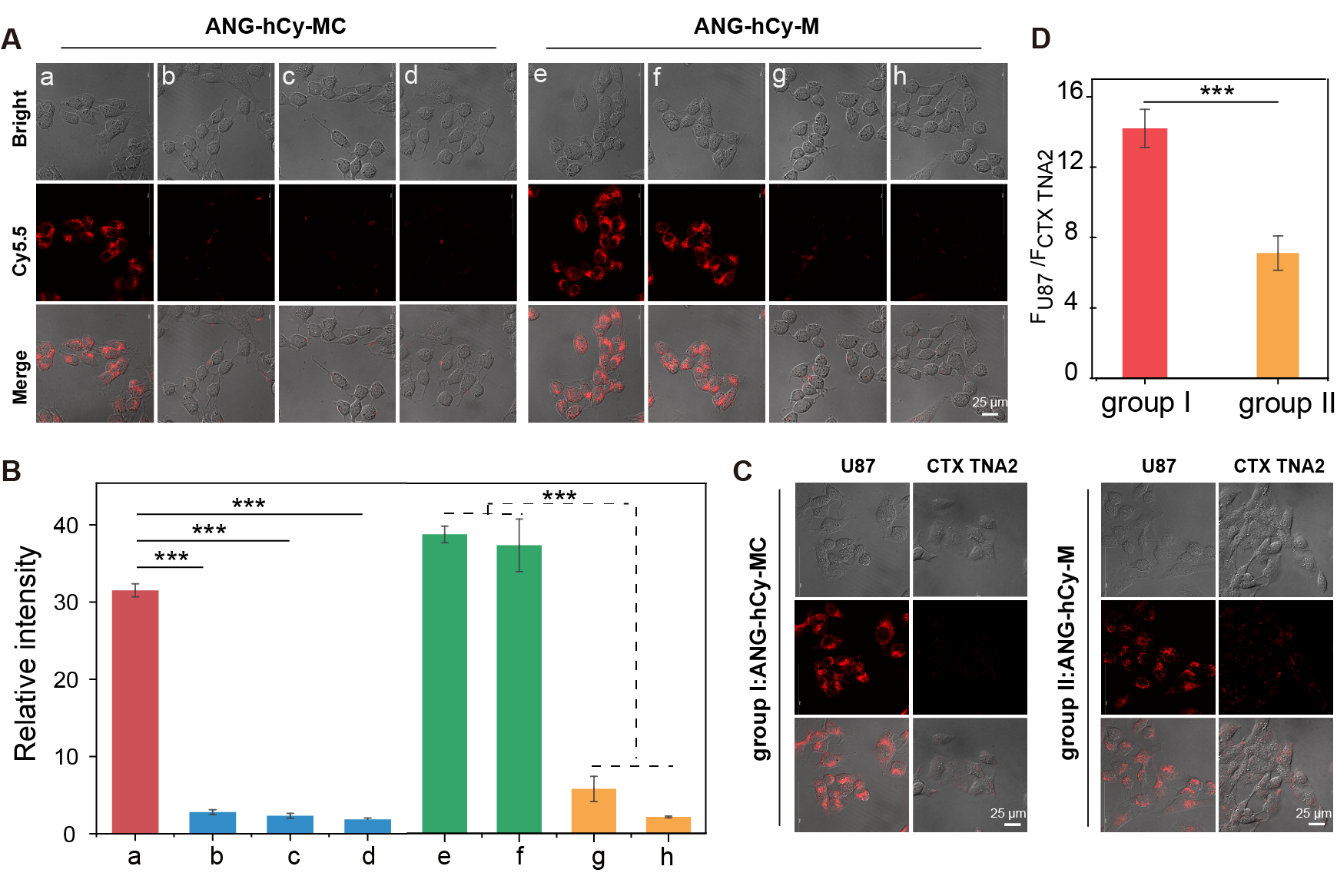


**Figure S10:** Fluorescence imaging in U87 cells. (A) Confocal fluorescence imaging of U87 cells using ANG-hCy-MC (10 µM) or ANG-hCy-M (10 µM). (a; e) Not treated with inhibitors U87 cells; (b; f) U87 cells were pre-incubated with CA-074 (100 µM ) for 2 h; (c; g) U87 cells were pre-incubated with CL (100 µM ) for 2 h; (d; h) U87 cells were pre-incubated with CA-074 (100 µM ) and CL (100 µM ) for 2 h. (B) Quantification of fluorescence intensity in Figure A. (C) Fluorescence images of U87 and CTX TNA2 cells treated with 10 µM ANG-hCy-MC or ANG-hCy-M. gruop I: (F _U87_/F _CTX TNA2_) of ANG-hCy-MC; gruop II: (F _U87_/F _CTX TNA2_) of ANG-hCy-M. (D) Fluorescence intensity ratio of ANG-hCy-MC or ANG-hCy-M in U87 and CTX TNA2 cells (F _U87_/F _CTX TNA2_) in Figure C. *λ_ex_*=640 nm, *λ_em_*=662-737 nm. Scale bar: 25 µm. (Data are expressed as means ± standard deviation (SD), n = 3, ***P < 0.001.)


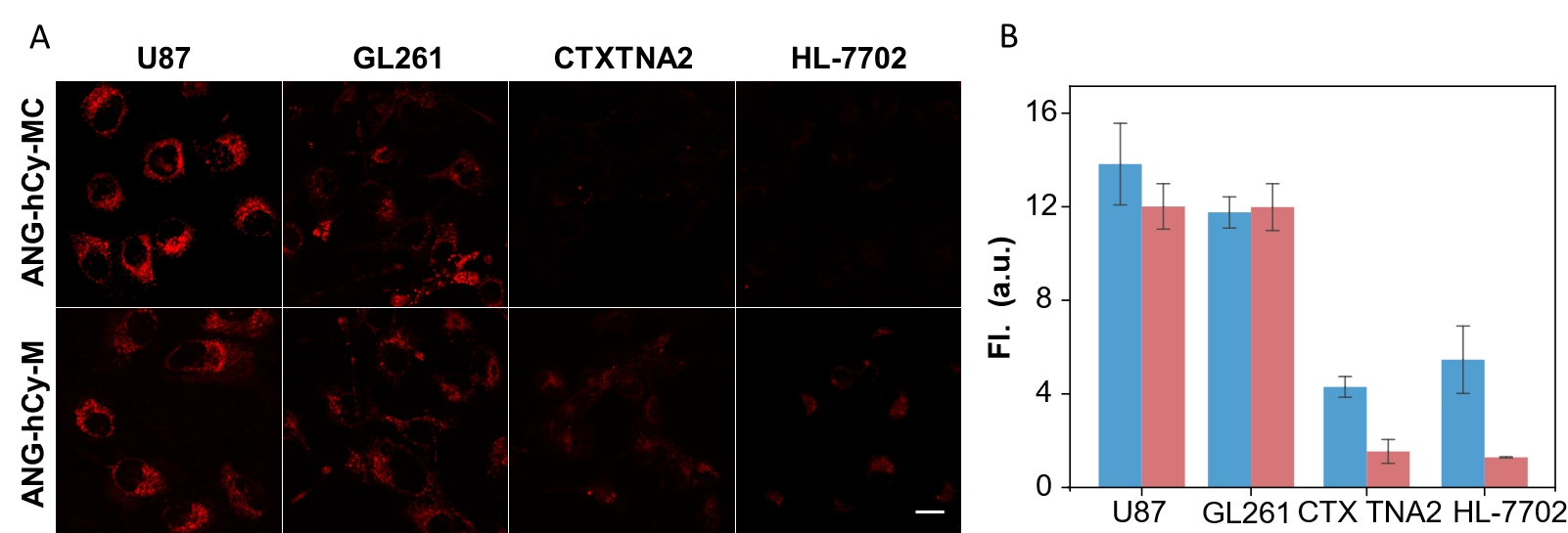


**Figure S11:** (A) Confocal imaging images of the single-lock probe ANG-hCy-M and the double-lock probe ANG-hCy-MC on different cells. (B) Quantification of fluorescence intensity in Figure A. *λ_ex_* =640 nm, *λ_em_* =662-737 nm. Scale bar: 25 µm.


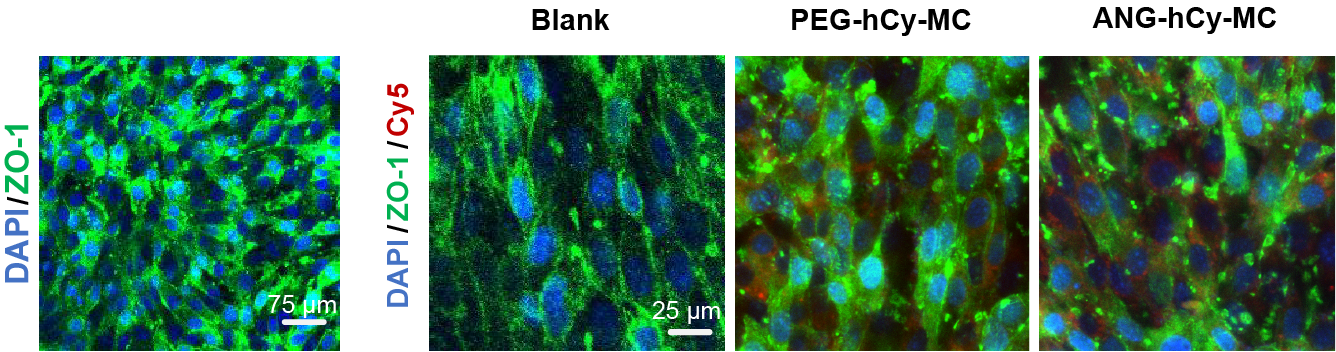


**Figure S12:** Immunofluorescence staining of the tight junction protein (ZO-1, green) in bEnd.3 cells, with DAPI (blue) counterstaining. Scale bar: 75 µm. Cy3: *λ_em_*=550-610nm.


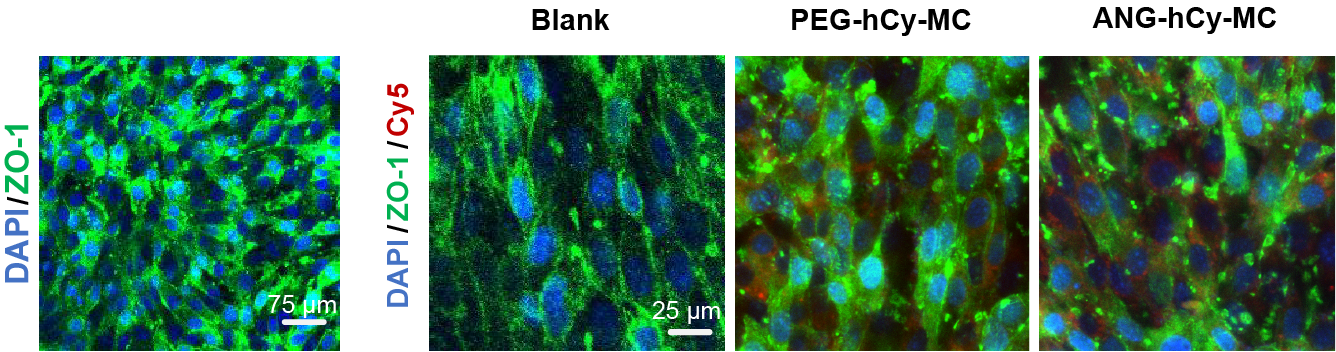


**Figure S13:** Representative confocal laser scanning micrograph of a single-layer cell of bEnd.3 in the upper row. Scale bar: 25 µm.


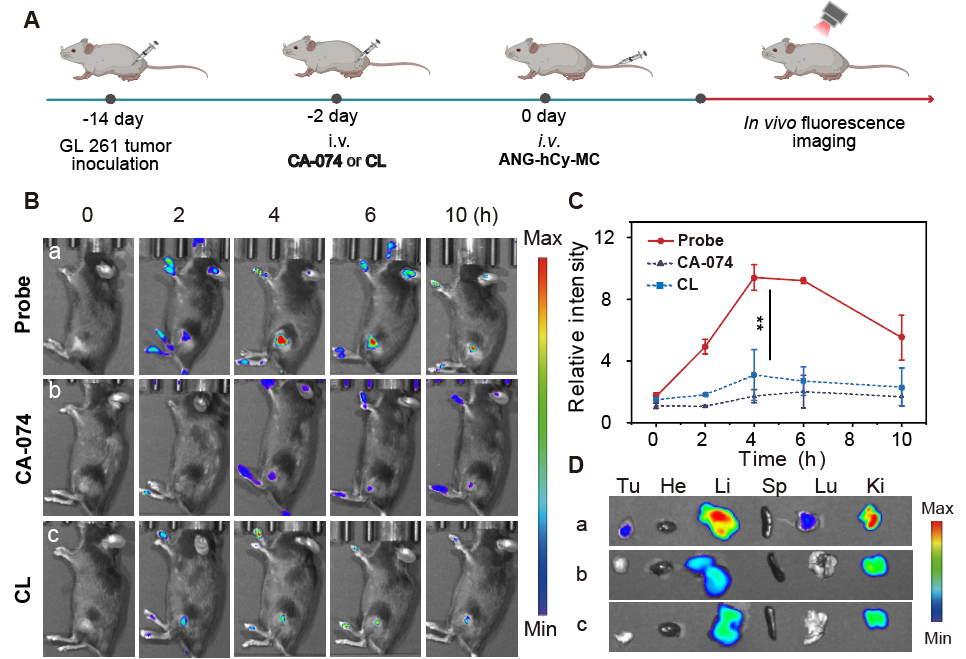


**Figure S14:** *In vivo* imaging of the GL261 tumor-bearing mouse. (A) Timeline of GL261 tumor-bearing mouse implantation and time-dependent imaging. (B) Fluorescence imaging of tumor-bearing mouse in different groups. a: ANG-hCy-M; b: ANG-hCy-M + CA-074; c: ANG-hCy-M + CL. (C) Quantification of fluorescence intensity in Figure B. (D) Organ *ex vivo* fluorescence imaging at 10 h after different groups. (Data are expressed as means ± standard deviation (SD), n = 3, **P < 0.01.) *λ_ex_*/*λ_em_* = 680/710 nm.


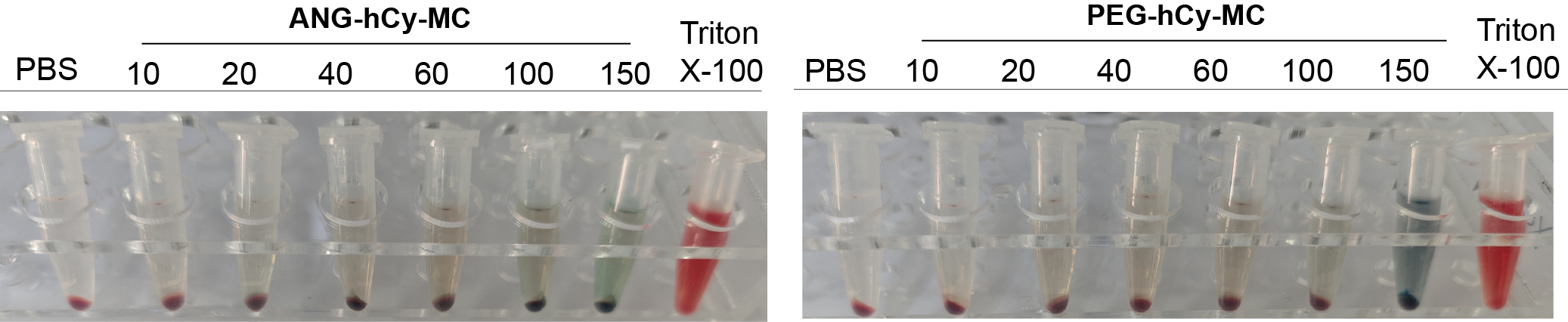


**Figure S15：**Hemolysis analysis of different concentrations of ANG-hCy-MC and PEG-hCy-MC (10, 20, 40, 60, 100, 150 μM).


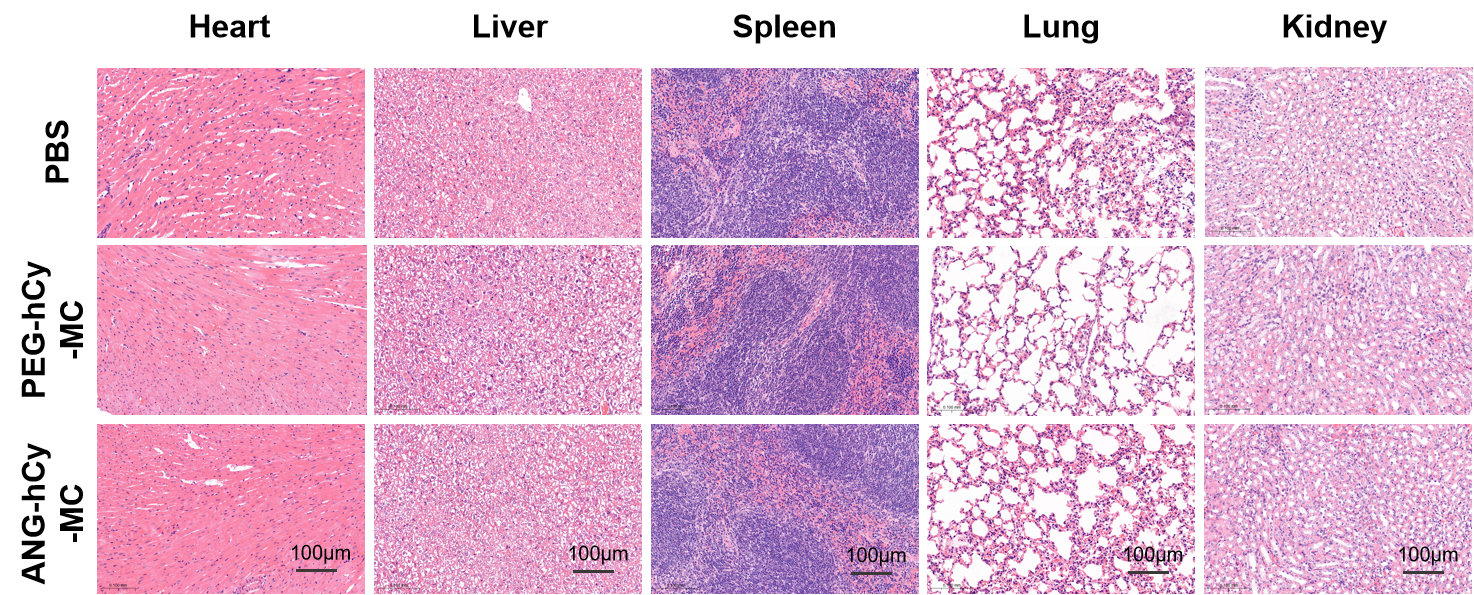


**Figure S16:** H&E staining of heart, liver, spleen, lung and kidney of mice injected with PBS, PEG-hCy-MC or ANG-hCy-MC for 24 h. Scale bar: 100 µm.


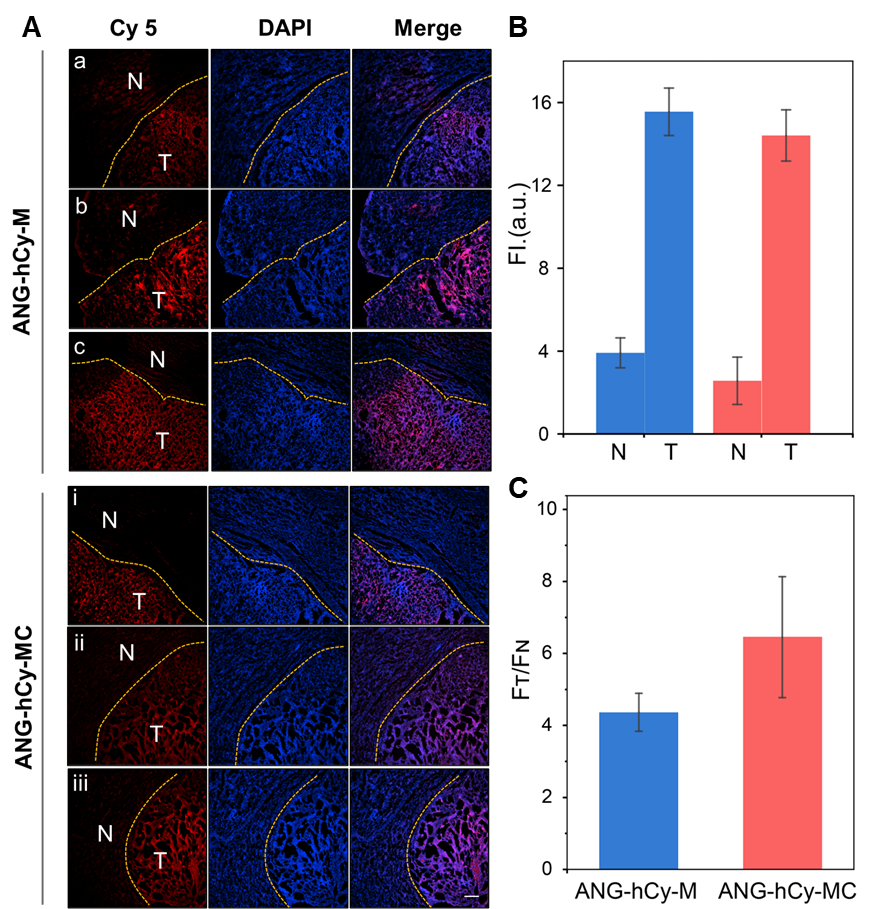


**Figure S17:** (A) Confocal imaging of single-lock probe ANG-hCy-M and double-lock probe ANG-hCy-MC on GBM tissue sections. (B) The fluorescence intensity of ANG-hCy-MC or ANG-hCy-M in the normal and tumor areas of the tissue sections (the blue bar graph represents ANG-hCy-M; the red bar graph represents ANG-hCy-MC). (C) Fluorescence intensity ratio of ANG-hCy-MC or ANG-hCy-M on GBM tissue sections (F_T_/F_N_) in Figure A. *λ_ex_*=640 nm, *λ_em_*=662-737 nm. Scale bar: 100 µm.


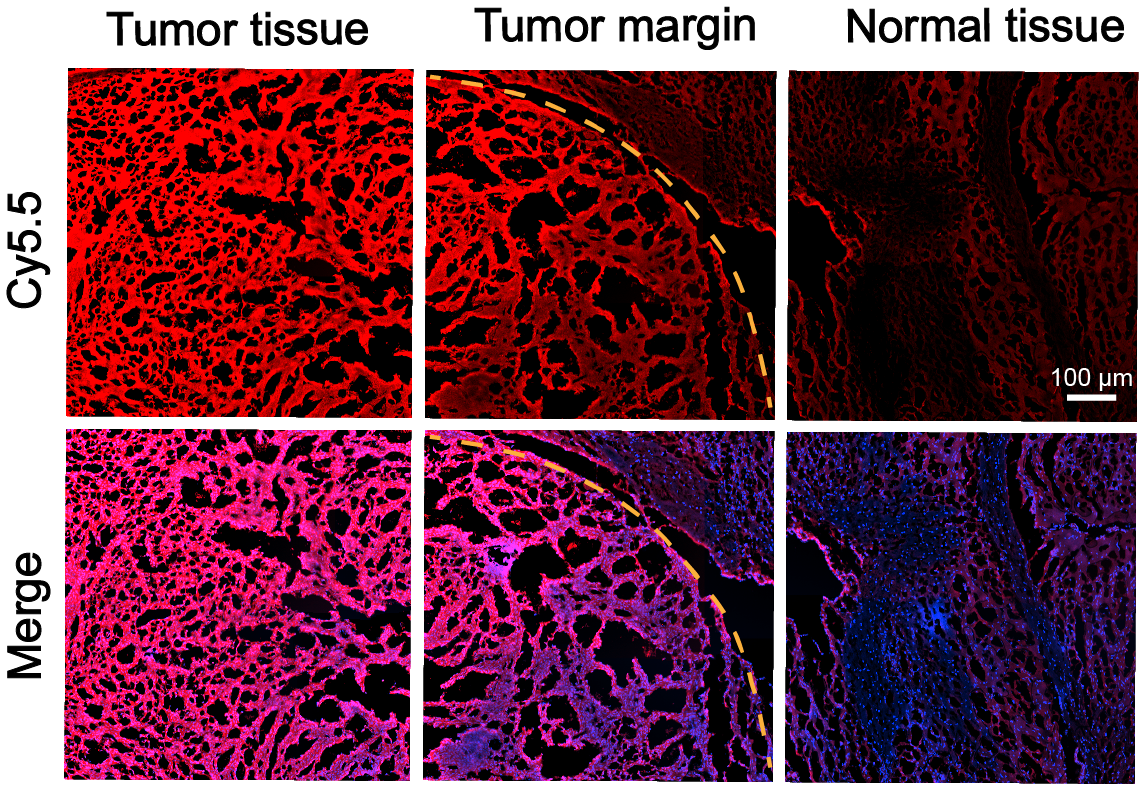


**Figure S18:** Confocal imaging of tumor tissues, tumor margins and normal tissues in GBM mouse sections. *λ_ex_*=640 nm, *λ_em_*=662-737 nm. Scale bar:100 µm.


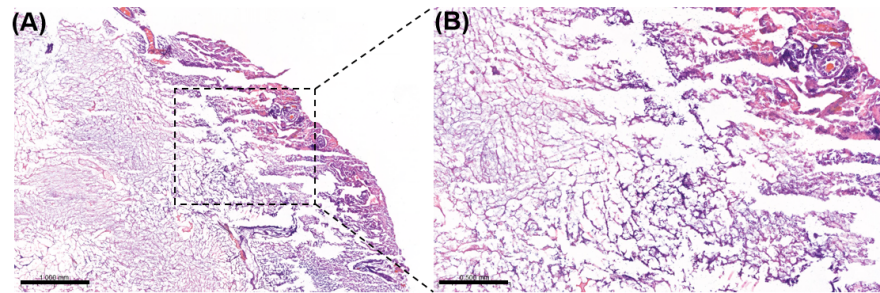


**Figure S19:** Clinical infiltrative margin of GBM. (A) The H&E staining of brain sections from clinical patients and (B) the enlarged images show that the boundary of GBM is not clear. (A) Scale bar:1mm (B) Scale bar: 500 µm.


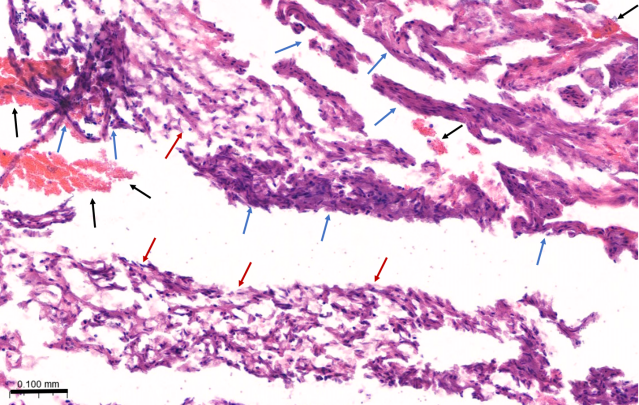


**Figure S20:** GBM region H&E staining analysis. (Blue arrow: dense area of tumor cells. Red arrow: glioma fiber area. Black arrow: area with vascular proliferation.) Scale bar: 100 µm.


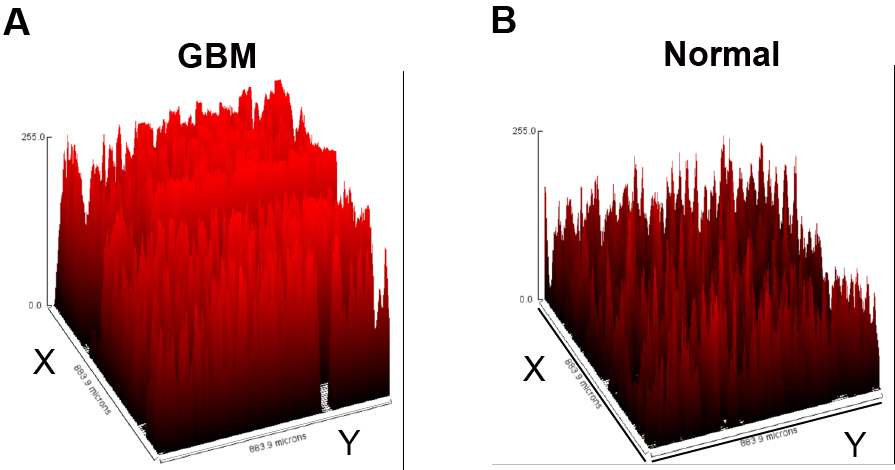


**Figure S21:** 3D surface reconstruction derived from Figure 6D.

1. **NMR and MS Spectra**

**Figure S22:** ^1^H NMR spectrum of hCy-OH in DMSO (400 MHz).


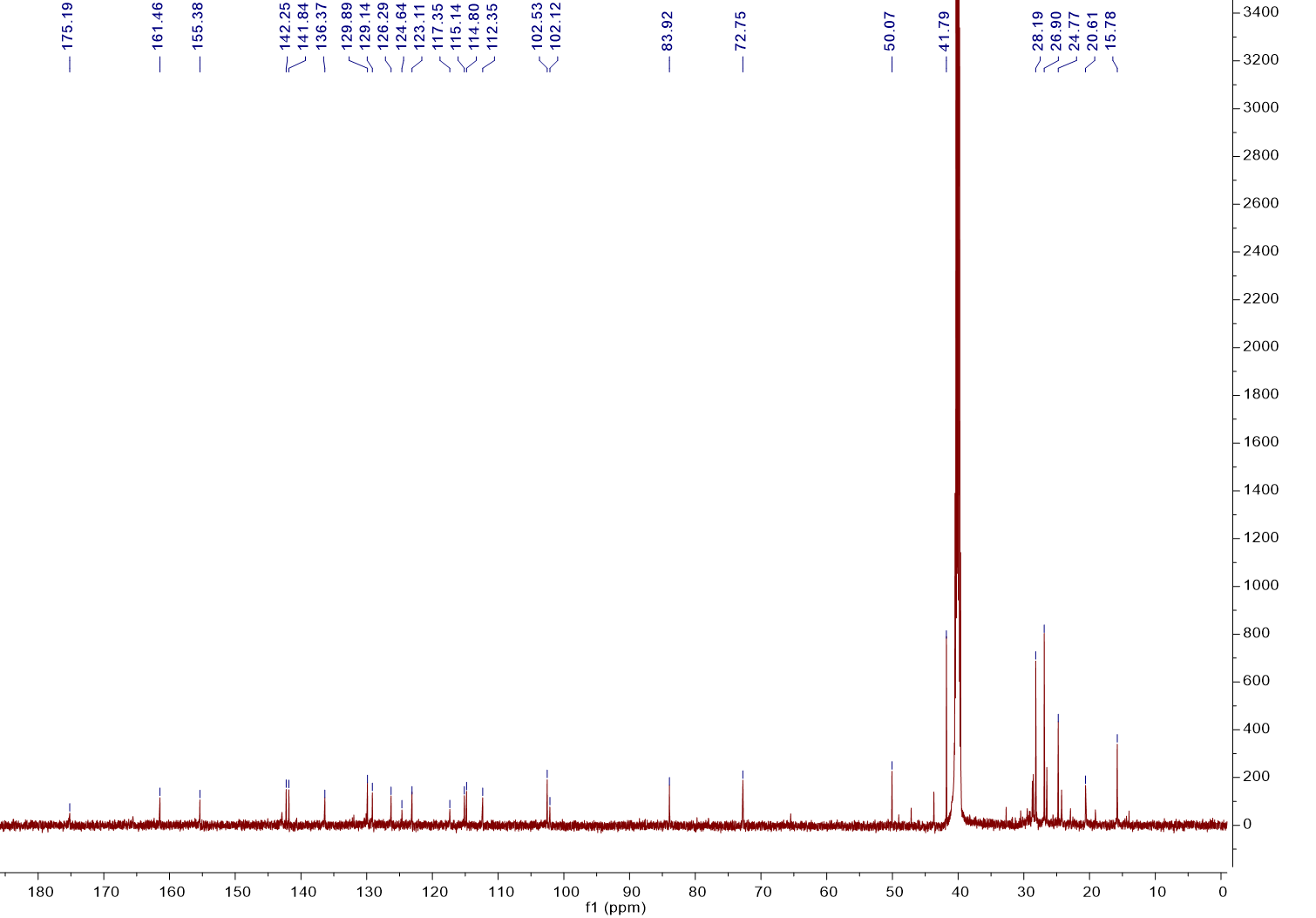


**Figure S23:** ^13^C NMR spectrum of hCy-OH in DMSO (400 MHz).

**Figure S24:** HRMS spectrum of hCy-OH.

**Figure S25:** ^1^H NMR spectrum of hCy-M in DMSO (400 MHz).

**Figure S26:** ^13^C NMR spectrum of hCy-M in DMSO (400 MHz).

**Figure S27:** HRMS spectrum of hCy-M.

**Figure S28:**^1^H NMR spectrum of hCy-MC in MeOH (400 MHz).


**Figure S29:** ^13^C NMR spectrum of hCy-MC in MeOH (400 MHz).

**Figure S30:** HRMS spectrum of hCy-MC.


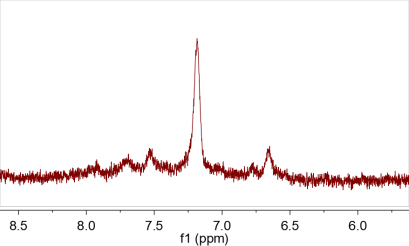

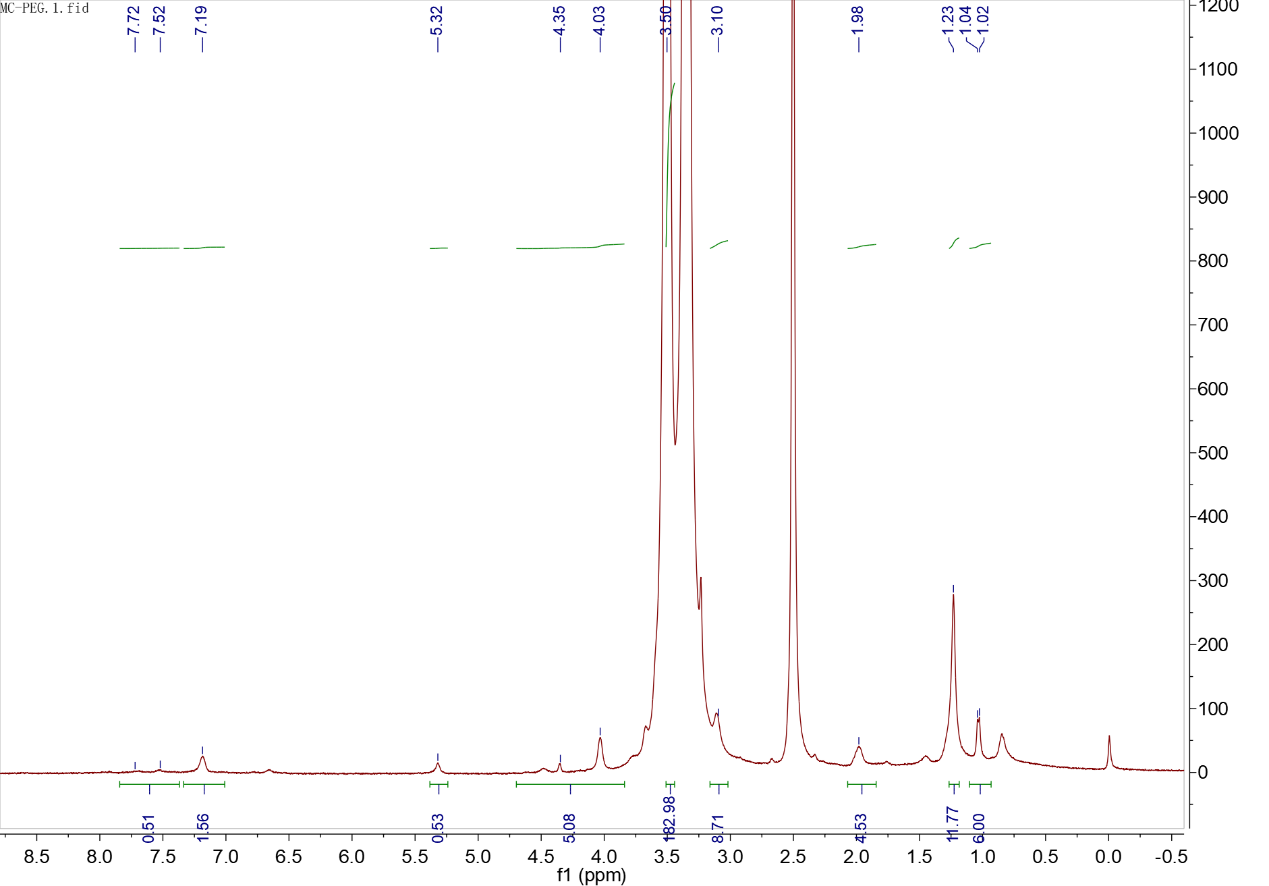


**Figure S31:**^1^H NMR spectrum of PEG-hCy-MC in DMSO (400 MHz).


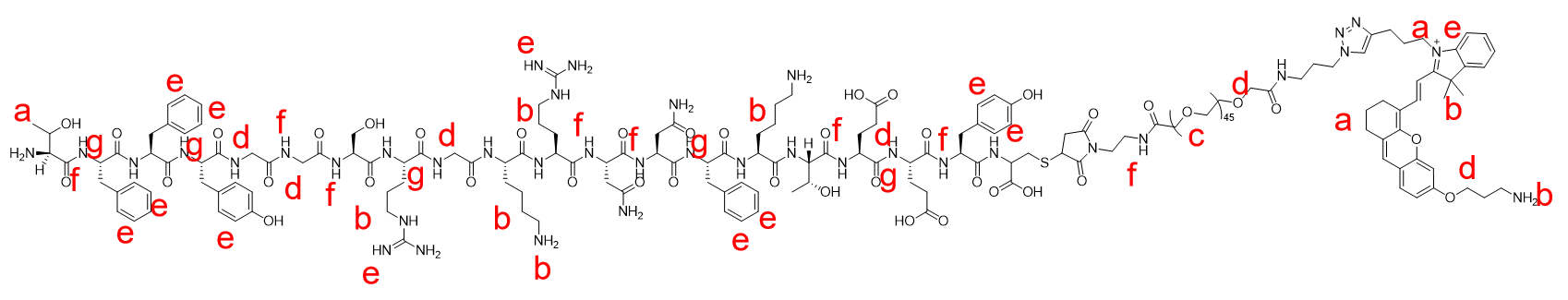


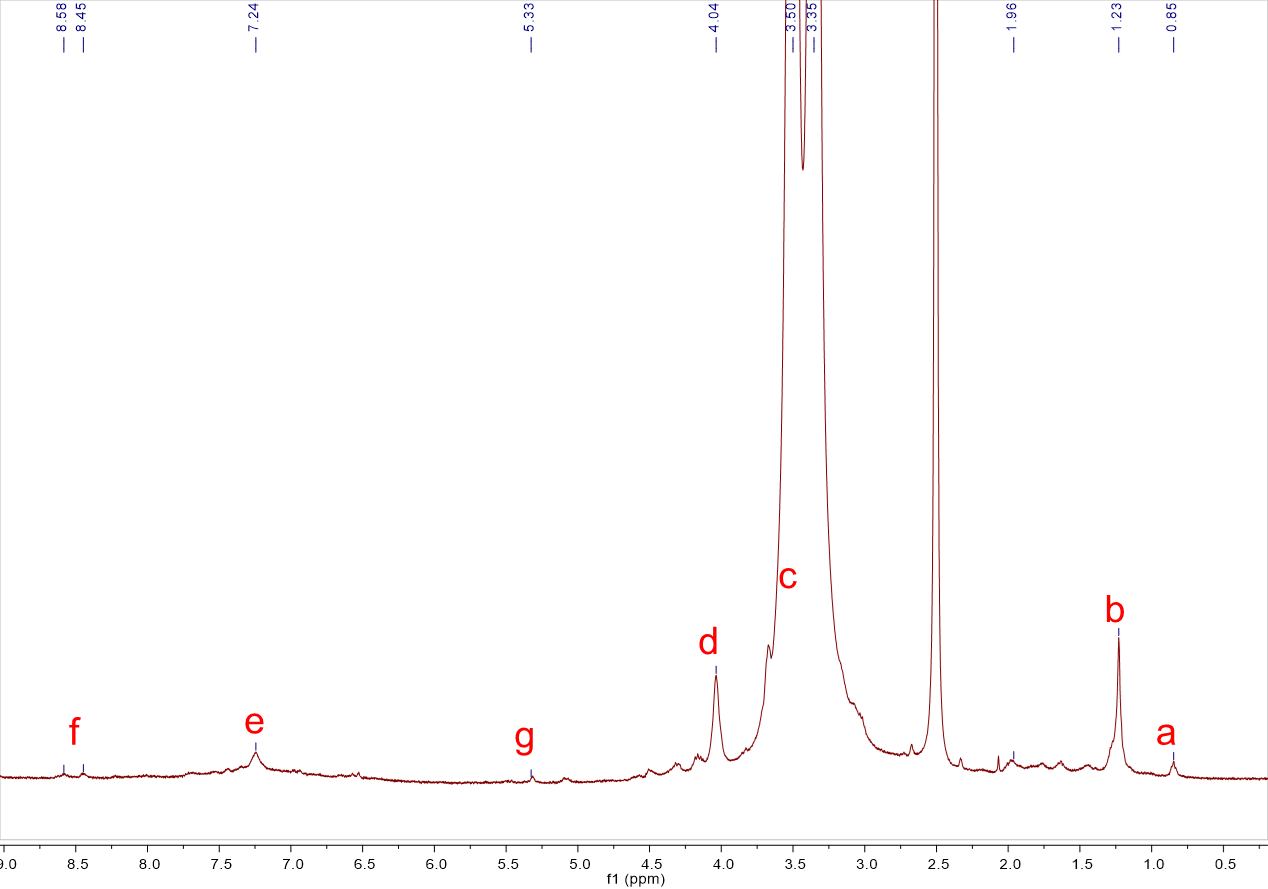


**Figure S32:**^1^H NMR spectrum of ANG-hCy-M in DMSO (400 MHz).


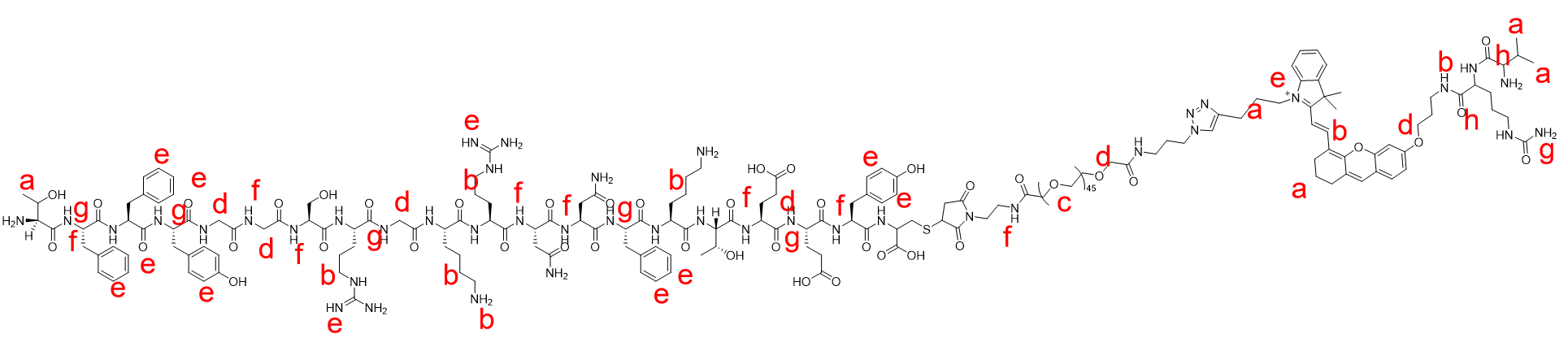


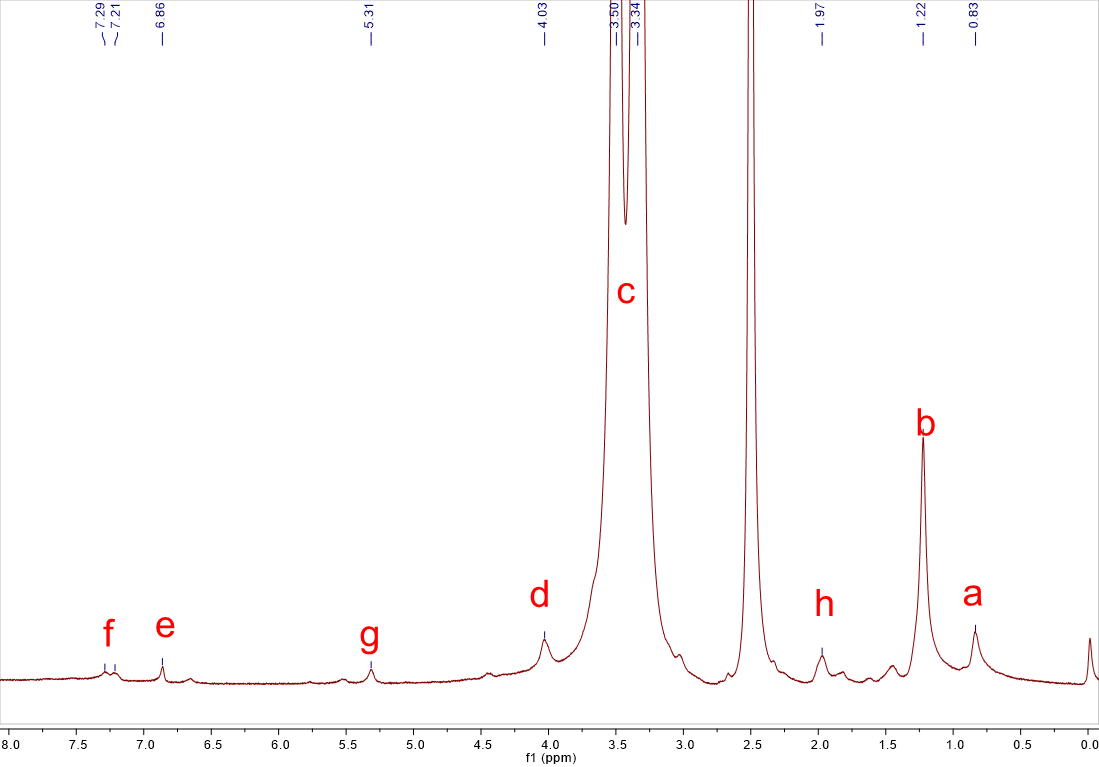


**Figure S33:**^1^H NMR spectrum of ANG-hCy-MC in DMSO (400 MHz).

**References**

[1] J. Fang, Y. Feng, Y. Zhang, A. Wang, J. Li, C. Cui, Y. Guo, J. Zhu, Z. Lv, Z. Zhao, "Alkaline phosphatase-controllable and red light-activated RNA modification approach for precise tumor suppression." *J. Am. Chem. Soc.* **2022,** *144*, 23061.
